# Bayesian inference with incomplete knowledge explains perceptual confidence and its deviations from accuracy

**DOI:** 10.1038/s41467-021-25419-4

**Published:** 2021-09-29

**Authors:** Koosha Khalvati, Roozbeh Kiani, Rajesh P. N. Rao

**Affiliations:** 1 Paul G. Allen School of Computer Science and Engineering, University of Washington, Seattle, WA USA; 2 Center for Neural Science, New York University, New York, NY USA; 3 Department of Psychology, New York University, New York, NY USA; 4 Neuroscience Institute, NYU Langone Medical Center, New York, NY USA; 5 Center for Neurotechnology, University of Washington, Seattle, WA USA

**Keywords:** Computational neuroscience, Decision, Network models

## Abstract

In perceptual decisions, subjects infer hidden states of the environment based on noisy sensory information. Here we show that both choice and its associated confidence are explained by a Bayesian framework based on partially observable Markov decision processes (POMDPs). We test our model on monkeys performing a direction-discrimination task with post-decision wagering, demonstrating that the model explains objective accuracy and predicts subjective confidence. Further, we show that the model replicates well-known discrepancies of confidence and accuracy, including the hard-easy effect, opposing effects of stimulus variability on confidence and accuracy, dependence of confidence ratings on simultaneous or sequential reports of choice and confidence, apparent difference between choice and confidence sensitivity, and seemingly disproportionate influence of choice-congruent evidence on confidence. These effects may not be signatures of sub-optimal inference or discrepant computational processes for choice and confidence. Rather, they arise in Bayesian inference with incomplete knowledge of the environment.

## Abstract (web summary)

A Bayesian framework based on partially observable Markov decision processes (POMDPs) not only predicts subjects’ confidence in a perceptual decision making task but also explains well-known discrepancies between confidence and choice accuracy as arising from incomplete knowledge of the environment.

## Introduction

Making decisions about hidden states of the environment based on noisy sensory information is critical for survival. Should an animal continue to graze after hearing a rustling sound? Was the sound due to a stalking predator or the wind? The outcome of such perceptual decisions is both a choice and an expectation of success known as confidence. Confidence plays a key role in guiding behavior in complex environments^1–4^ and is often critical for modeling behavior and understanding its neural mechanisms in such environments^5–10^. However, unlike sensory choices and their accuracy that are usually easy to measure, confidence is a subjective quality difficult to measure reliably, unless special experimental procedures are employed^2,11–17^. Experiments that make such measurements have often revealed systematic discrepancies between subjective confidence reports and experimentally measured accuracy^15,16,18–22^. These discrepancies have been occasionally interpreted as evidence for suboptimality of the decision-making process or for disparate processes for computing choice and confidence. Contrary to those interpretations, we show that a Bayesian framework with optimal inference but incomplete knowledge about the environment can explain choice accuracy, confidence, and their discrepancies in experimental measurements.

Our model extends Partially Observable Markov Decision Processes (POMDPs)^23^, which assume that subjects optimize a reward function by adjusting their beliefs about stimulus identity and the best choice based on two factors: sensory observations and prior knowledge about environmental states^24–26^, which are learned from past experience. The model enables us to simulate temporal update of belief for a sequence of sensory observations. These belief updates generate explicit links between the decision maker’s confidence and choice accuracy. We demonstrate the precision of our predictions about choice confidence by testing them on monkeys performing a direction discrimination task with post-decision wagering^2^, where both choice accuracy and confidence were measured.

In addition to explicitly linking confidence and accuracy, our model explains well-known discrepancies between these two measurements. Some discrepancies arise in an optimal decision-making process when the decision maker has incomplete knowledge about the environment and needs to resolve uncertainties about the reliability of observations. Others seem to exist from an experimenter’s perspective because the exact information used by the subject is hidden to the experimenter. Our POMDP model explains commonly observed discrepancies between accuracy and confidence such as the hard-easy effect^6,19,27^, higher confidence with increased variability of sensory observations despite reduction of accuracy^16,28^, different confidence ratings in simultaneous versus sequential reports of choice and confidence^12,27,29^, discrepancy between sensitivities of accuracy and confidence (${d}^{\prime}$ vs. meta-${d}^{\prime}$)^30,31^, and the seemingly larger effect of choice-congruent observations on confidence reports^15,20^.

We conclude by showing that the Bayesian inference component of our POMDP model can be implemented by the neural mechanisms that integrate evidence toward a decision bound, consistent with drift diffusion models (DDMs)^32^ or more generally, models based on bounded-accumulation of evidence. The POMDP model commits to a choice when the value of the expected improvement of accuracy with new observations is less than the cost of making those observations. We show that this termination criterion uniquely maps to a time-varying decision bound for integration of evidence in the DDM (shown also by Huang and Rao^26^). Such time-varying bounds match past behavioral studies^33,34^ and can be implemented by the urgency signals observed in electrophysiological recordings^9,35,36^. Overall, the neural implementation of inference and choice in our POMDP framework is both simple and plausible.

## Results

**Fig. 1:**
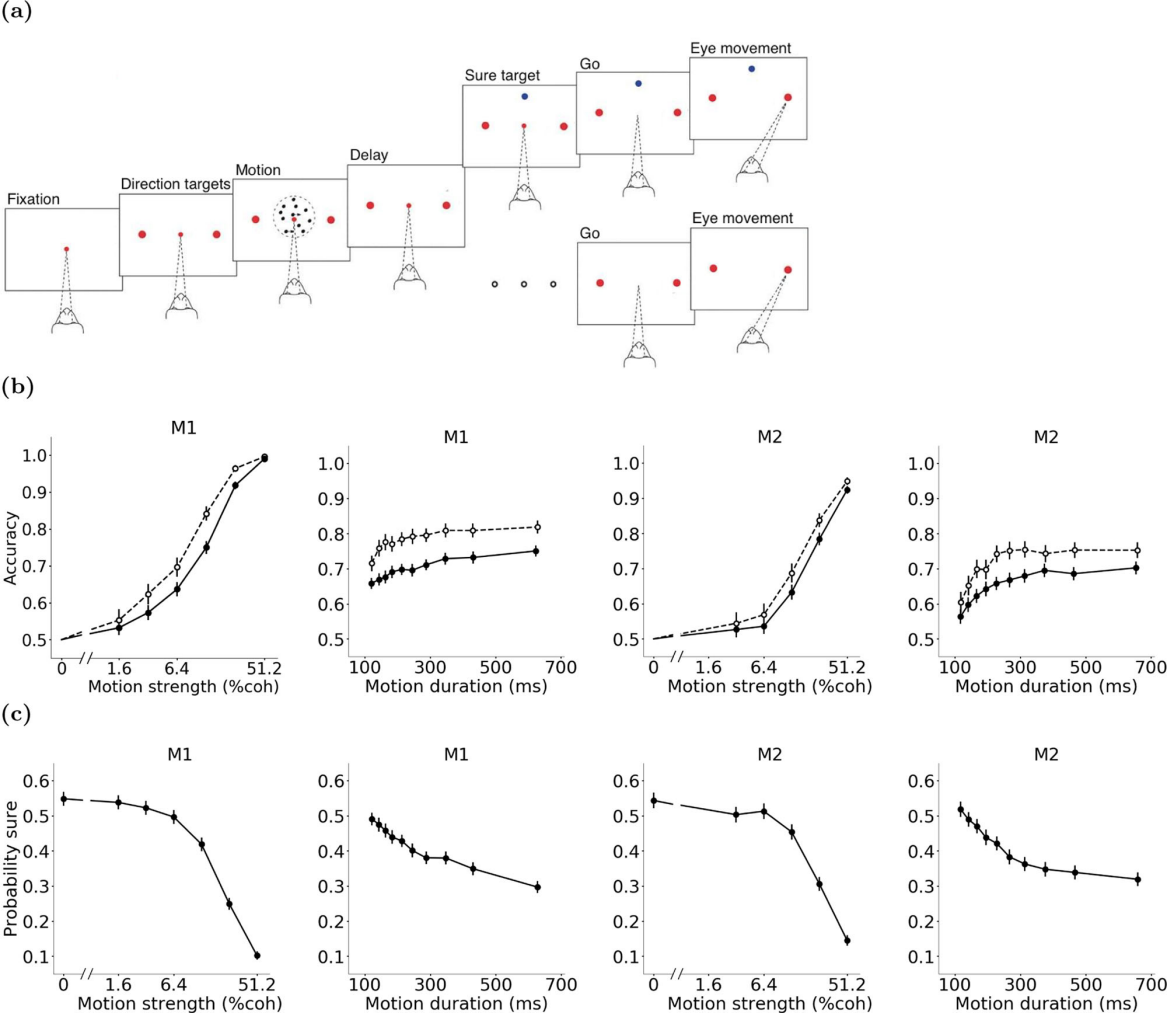
Motion direction discrimination with post-decision wagering. **a** Task design. On each trial, monkeys viewed a patch of randomly moving dots and made a decision about the net direction of motion. Stimulus strength and duration varied randomly from trial to trial. On half of the trials, only the right and left direction targets were shown (large red dots). The motion stimulus was followed by a delay period. The central fixation point (small red dot) disappeared at the end of the delay (Go cue), instructing the monkey to report perceived motion direction with a saccadic eye movement to one of the two direction targets. Choosing the correct target yielded a large reward, whereas choosing the incorrect target resulted in a short timeout. On the other half of the trials, a third target (sure target, shown as a blue dot) appeared on the screen during the delay period. Choosing this target after the Go cue yielded a guaranteed but smaller reward than choosing the correct direction target. **b** Accuracy as a function of motion strength and duration for the two monkeys (M1 and M2). For the motion strength plots, trials are pooled across all durations. For the motion duration plots, trials are pooled across all strengths. Solid lines show the accuracy on trials where the sure target was not presented (M1 motion strength plot: *n* = 6174 ± 112 per data point; M1 motion duration plot: *n* = 3720 per data point; M2 motion strength plot: *n* = 5035 ± 56 per data point; M2 motion duration plot: *n* = 2530 per data point). Dashed lines show the accuracy on trials where the sure target was shown but the monkey chose one of the high-stakes direction targets (M1 motion strength plot: *n* = 4148 ± 1314 per data point; M1 motion duration plot: *n* = 2261 per data point; M2 motion strength plot: *n* = 3376 ± 917 per data point; M2 motion duration plot: *n* = 1557 per data point). **c** Probability of choosing the sure target for different motion strengths and durations for monkeys M1 and M2 (M1 motion strength plot: *n* = 6195 ± 113 per data point; M1 motion duration plot: *n* = 4315 per data point; M2 motion strength plot: *n* = 5081 ± 79 per data point; M2 motion duration plot: *n* = 3031 per data point). Error bars indicate standard error of the mean (s.e.m.).

We developed and tested our model using behavioral data from monkeys performing a direction discrimination task with post-decision wagering (Fig. 1a)^2^. On each trial, monkeys observed a patch of randomly moving dots^37^ and decided about the net direction of motion. The difficulty of the decision was varied randomly from trial to trial by changing the percentage of coherently moving dots (the “motion strength” or “coherence”) and the duration of the motion stimulus (Fig. 1b). The stimulus was followed by a delay period and at the end of the delay, the fixation point disappeared (Go cue), signaling the monkey to report its choice with a saccadic eye movement. On a random half of trials, the monkey was given only the right and left direction targets. Choosing the correct motion direction (right target for rightward motion and left target for leftward motion) resulted in a large reward (a large drop of juice) but choosing the incorrect target resulted in no reward and a short timeout. On the other half of trials, the monkey was offered a third target, in addition to the direction targets, in the middle of the delay period. This third target was a sure-bet option. The monkey could choose either the direction targets or the sure-bet after the Go cue. Choosing the sure-bet target guaranteed reward but the magnitude of the reward (volume of the juice) was smaller than that for choosing the correct direction target.

An optimal decision maker who desires to earn more reward and maximize utility should choose the risky, high-paying direction targets when confident about motion direction and the sure-bet option when doubtful about the correct direction. Monkeys showed a similar behavioral pattern. They chose the sure-bet option more often on more difficult trials, where motion strength was low or motion duration was short (Fig. 1c). Further, when they ignored the sure-bet option and chose the high-stakes direction targets, their accuracy was higher compared to the trials with similar difficulty without the sure-bet option when they had to choose one of the direction targets (trials without sure-bet target; Fig. 1b). These results indicate the presence of a mechanism for assessment of expected decision outcome (confidence), and reliance on this mechanism for guiding the opt-out behavior.

### Modeling perceptual decision making with a POMDP

In perceptual decision-making tasks, an ideal observer would infer hidden states of the environment based on a sequence of sensory observations to gain the maximum possible reward utility. This problem can be solved using the general framework of POMDPs, which combines Bayesian inference of hidden states with expected reward maximization^23–25,38–40^. Formally, a POMDP is a tuple (*S*, *A*, *Z*, *T*, *O*, *R*) where *S* and *Z* are two sets containing the states of the environment and observations, respectively. *A* is the set of possible actions. *T* is a transition function that represents the probability of entering a state *s* from a state $s^{\prime}$ after performing an action *a*: $T(s,s^{\prime} ,a)=P(s| s^{\prime} ,a)$. Note that the environment is assumed to be Markovian, meaning that the next state depends only on the current state and current action. *O* is the observation function, determining the probability of making an observation *z* given a state *s*, i.e., *O*(*s*,*z*) = *P*(*z*∣*s*). The current state of the environment is not known to the decision maker and needs to be inferred based on the history of observations and actions. A POMDP starts with a prior probability distribution over states of the environment, known as the *initial belief b*_0_, and infers the posterior probability distribution (*belief b*_*t*_) of states after each action and observation:

1 $${b}_{t}(s)\propto P({z}_{t}| s)\mathop{\sum}\limits_{s^{\prime} \in S}P(s| s^{\prime} ,{a}_{t-1}){b}_{t-1}(s^{\prime} )$$

Finally, *R* is the reward utility function: *R*(*s*_*t*_, *a*_*t*_). We emphasize reward utility instead of reward size, as the model optimizes the benefit of reward (utility) and the utility of reward does not grow linearly with reward size for a wide range of tasks and behaviors. A policy *π* is a mapping from belief states (probability distributions over states) to actions. The optimal policy π^*^ is a policy that maximizes the total expected reward utility. In a task where the maximum number of steps is *H*, the optimal policy is given by:

2 $${\pi }^{* }=\mathop{{{{{{{{\rm{arg}}}}}}}}\ {{{{{{{\rm{max}}}}}}}}}\limits_{\pi }\mathop{\sum }\limits_{t=0}^{H}E[R({s}_{t},{a}_{t})| {b}_{0},\pi ].$$

We next define within the POMDP framework the concepts of accuracy and confidence as used in the perceptual decision making literature^18^. Similar to how accuracy is calculated for experimental data, choice accuracy for the POMDP model can be defined as the fraction of trials in which the choice leads to reward. Additionally, following previous definitions of confidence as the expected likelihood of success in symmetric two-alternative choice tasks where only one action leads to a reward^2,6,12,18,40–42^, we define confidence as the expectation of the model that its selected action *a*_*t*_ maximizes utility, i.e.:

3 $$Confidence=P\left({a}_{t}=\mathop{{{{{{{{\rm{arg}}}}}}}}\ {{{{{{{\rm{max}}}}}}}}}\limits_{a}R({s}_{t},a)| {b}_{t},\pi \right).$$

To be consistent with other studies, we limit the use of confidence to actions that terminate the process of decision making (e.g., the two direction choices in the motion direction discrimination task).

### POMDP model of the direction discrimination task

**Fig. 2:**
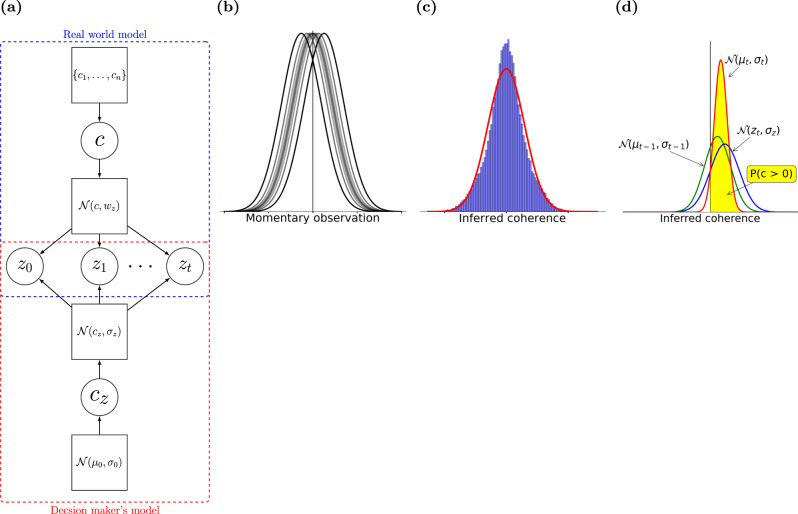
The POMDP model of the direction discrimination task. **a** Our modeling framework depicting the real world generative model and the decision maker’s model. In each trial, the state (here signed coherence *c*) is generated from a prior (here a discrete set selected by the experimenter). Given a value for *c* for a trial, all of the observations *z*_*i*_ in that trial are independent samples generated from an observation function (${{{{{{{\mathcal{N}}}}}}}}(\mu =c,{w}_{z})$). While the decision maker has full access to the observations, the prior distribution and the observation function are not known. Our POMDP model estimates these two distributions as ${{{{{{{\mathcal{N}}}}}}}}({\mu }_{0}=0,{\sigma }_{0})$ and ${{{{{{{\mathcal{N}}}}}}}}({c}_{z},{\sigma }_{z})$ where *σ*_0_ and *σ*_*z*_ are learned from data (see text), and *c*_*z*_ is the coherence which is estimated by the subject using the belief ${{{{{{{\mathcal{N}}}}}}}}({\mu }_{t},{\sigma }_{t})$ as described in the text. **b** Probability distribution of momentary observations for a motion coherence *c* is modeled as a Gaussian distribution with mean *μ* = *c* and variance ${w}_{z}^{2}$. There are multiple Gaussian distributions for different motion coherences. Positive and negative observations indicate rightward and leftward motion directions, respectively. **c** The distribution of inferred coherence across all trials provides the initial belief state of the POMDP model (blue histogram) at the beginning of each trial. The initial belief is approximated by a Gaussian function (red curve). **d** The POMDP model sequentially updates its belief about the motion coherence based on new observations. Combining the belief at time *t* − 1 (green distribution) with the acquired observation from the stimulus at time *t* (blue distribution) results in a new belief at *t* (red distribution). The expected likelihood that the rightward choice is correct is the area under the updated belief distribution for positive sensory evidence (yellow region).

The motion direction discrimination task has previously been modeled using the POMDP framework^24,26,40,43^. However, in these models, the subject’s confidence was either not modeled^24,26^ or was obtained assuming the subject had an exact generative model of the task^40,43^. Such knowledge, however, is unlikely in most natural contexts and common task designs. For example, in the direction discrimination task, subjects face a mixture of stimulus difficulties across trials. They neither know the exact generative function for the stimulus on each trial nor the exact set of motion strengths used in the experiment. To model this situation, we use the framework depicted in Fig. 2a consisting of two models: the real model of the environment and the learned model used by the decision maker (their internal model).

Following previous models^24,43^, we define the hidden state of the environment for our POMDP to include both the unknown direction and unknown coherence, combined into a single real-value which we call "signed motion coherence" *c*: positive values of the signed motion coherence indicate rightward motion and negative values indicate leftward motion^44^. Specifically, the momentary observations *z*_*t*_ at times *t* for a trial with signed coherence *c* are modeled as samples independently drawn from a Gaussian distribution, ${{{{{{{\mathcal{N}}}}}}}}(c,{w}_{z})$, with mean *μ* = *c* and variance ${w}_{z}^{2}$ (Fig. 2b).

The two main actions of our POMDP model are committing to direction right or direction left. Also, action "observe” makes the next observation available to update the model’s belief about *c*. Finally, the action of choosing the sure-bet option is available during the delay period on half of the trials. The decision maker gets *r*_*right*_ as the reward utility for committing to direction right if and only if the direction of the hidden state is right (*c* > 0). *r*_*left*_ is the reward utility given to the decision maker by committing to direction left if and only if the direction of the hidden state is left (*c* < 0). Choosing the sure-bet option, if available, always yields reward utility of *r*_*sure*_.

The POMDP model begins each trial with a prior belief about the signed coherence of the trial. Subjects are not explicitly informed about the exact set of discrete motion coherence levels used in the experiment. They only experience largely overlapping distributions of motion energies on different trials^45^. Therefore, it is most realistic to consider that the model’s prior spans a continuous domain, obtained from observations across all trials with various coherence levels and durations. Because the logarithmic spacing of the discrete motion coherences used in the experiments (0, 1.6, 3.2, 6.4, 12.8, 25.6, 51.2%) causes the mass of the prior distribution to be largely concentrated in its central peak around 0, our POMDP model uses a Gaussian approximation to this prior distribution, ${{{{{{{\mathcal{N}}}}}}}}(0,{\sigma }_{0})$ (Fig. 2c).

Starting with a Gaussian prior (initial belief) ${b}_{0}={{{{{{{\mathcal{N}}}}}}}}({\mu }_{0}=0,{\sigma }_{0})$, the model iteratively updates its belief about the hidden state of the environment, i.e., the signed motion coherence *c*, following each observation, *z*_*t*_, drawn from the distribution ${{{{{{{\mathcal{N}}}}}}}}(c,{w}_{z})$ at time step *t* (Fig. 2d). To be able to update the belief, knowledge of the true observation variance, ${w}_{z}^{2}$, is required. However, ${w}_{z}^{2}$ is unknown to the model. Therefore, we use ${\sigma }_{z}^{2}$ to denote the model’s learned observation variance. This means that the model assumes *z*_*t*_ is drawn from the Gaussian likelihood function $P({z}_{t}| c)={{{{{{{\mathcal{N}}}}}}}}({z}_{t};c,{\sigma }_{z})$. A Gaussian prior and a Gaussian likelihood function together result in a Gaussian posterior^43,46^ (Fig. 2d) for *c* given by:

4 $${b}_{t}=	\; P(c| {z}_{1},\ldots ,{z}_{t})={{{{{{{\mathcal{N}}}}}}}}({\mu }_{t},{\sigma }_{t})\\ {\mu }_{t}=	\; \frac{{\sigma }_{t-1}^{2}{z}_{t}+{{\sigma }_{z}^{2}\mu }_{t-1}}{{\sigma }_{t-1}^{2}+{\sigma }_{z}^{2}}=\frac{{\sigma }_{z}^{-2}}{t{\sigma }_{z}^{-2}+{\sigma }_{0}^{-2}}\mathop{\sum }\limits_{j=1}^{t}{z}_{j}\quad {\sigma }_{t}^{2}=\frac{{\sigma }_{t-1}^{2}{\sigma }_{z}^{2}}{{\sigma }_{t-1}^{2}+{\sigma }_{z}^{2}}=\frac{1}{t{\sigma }_{z}^{-2}+{\sigma }_{0}^{-2}}$$

Since the reward only depends on choosing the correct motion direction, the POMDP model’s choice depends on *μ*_*t*_, and consequently $\mathop{\sum }\nolimits_{j = 1}^{t}{z}_{j}$, being larger than zero for choosing the rightward direction and less than zero for choosing the leftward direction. A random choice is made in the unlikely event that *μ*_*t*_ is exactly equal to 0. Moreover, according to equation (3), the model’s confidence is the posterior probability of the chosen direction, which is the sum of the posterior probabilities over all motion coherences in that direction, i.e., Φ(*μ*_*t*_/*σ*_*t*_) when *μ*_*t*_ ≥ 0 and Φ(−*μ*_*t*_/*σ*_*t*_) when *μ*_*t*_ < 0, where Φ(*x*) denotes the standard normal cumulative distribution function^43,47^.

**Fig. 3:**
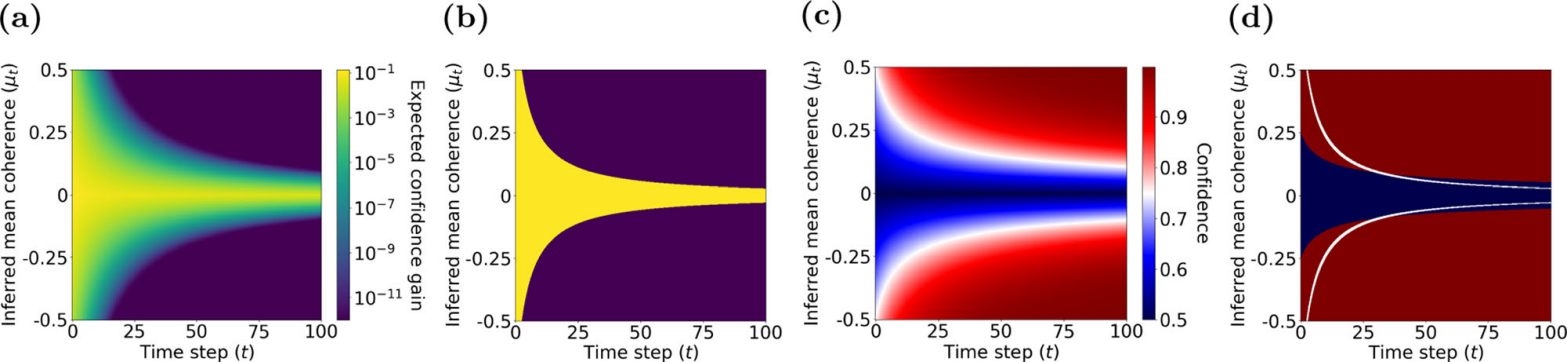
Computation of choice and confidence in the POMDP model of the direction discrimination task. **a** The expected confidence gain for a new observation as a function of inferred mean coherence, *μ*_*t*_, and elapsed time, *t*. **b** An example POMDP decision policy when new observations are associated with a constant cost. The yellow area represents the belief states where the optimal action is to continue observing. The purple area represents the belief states where the POMDP model terminates and commits to a choice. **c** Confidence as a function of inferred mean coherence, *μ*_*t*_, and time, *t*. **d** The ratio of reward utilities for sure-bet and correct direction choices determines the POMDP policy for choosing the sure-bet option. The policy for sure-bet can be illustrated as phase boundaries in the confidence plot of **c**. The blue region denotes combinations of inferred coherence and time for which the model would choose the sure-bet target. The red region denotes (*μ*_*t*_, *t*) for which direction targets are chosen. Thresholds for separating low and high confidence ratings are thus the boundaries between blue (low confidence) and red (high confidence) regions. Solid white lines show the two decision termination bounds where the model stops gathering more observations and commits to a decision. In these simulations *σ*_*z*_ = 2.0, *σ*_0_ = 1.0, and the utility ratio = 0.63.

The POMDP approach can easily model termination of the decision-making process and commitment to a choice by assigning a cost (negative utility) to observation gathering and belief update (via the action “observe”)^24^. Moreover, because the hidden state does not change with actions within a trial in the motion discrimination task, a one-step look-ahead search^38^ is adequate to determine the optimal decision policy for non-decreasing observation costs over time (instead of computing the total expected reward utility to the end of the trial; see the proof in Methods). The model halts new observations when the expected increase in confidence is less than the ratio of the cost of an observation and the reward utility for correct choice. The expected increase in confidence after one more observation depends on the current belief and the probability distribution of the next observation according to the model. Specifically, when the current belief is ${{{{{{{\mathcal{N}}}}}}}}({\mu }_{t},{\sigma }_{t})$, the model assumes that the next observation is a sample from ${{{{{{{\mathcal{N}}}}}}}}({\mu }_{t},{\sigma }_{z})$, where ${\sigma }_{z}^{2}$ is the learned observation variance. Fig. 3a shows the expected increase in confidence for a new observation as a function of two key variables: the inferred *μ*_*t*_ and the elapsed time. The expected increase in confidence from new observations is higher earlier in the trial and for smaller inferred mean coherence, *μ*_*t*_.

A constant observation cost over time, if present, would give rise to a stopping criterion that matches an iso-gain contour. These contours would effectively implement a time-varying bound on *μ*_*t*_ for each motion direction (an upper bound and a lower bound). Fig. 3b shows these collapsing bounds for a cost of 10^−3^ per observation (in our case, per 10 ms) when the reward utility for a correct direction choice is set to 1. A policy for termination of observations is especially critical in reaction-time (RT) tasks where subjects have to decide when to initiate a response. However, a termination policy could exist even in tasks where stimulus duration is controlled by the experimenter, causing early termination of the subject’s decision-making process before stimulus offset, especially in long and easy trials^45,48^.

The reward utility maximization principle also determines the choice when the sure-bet option is available. As the reward for the sure-bet option is guaranteed, the POMDP model compares the expected reward utility for choosing each direction with the reward utility for the sure-bet option in order to pick the final action:

5 $${a}_{t}=\left\{\begin{array}{ll}left&{b}_{t,left}\cdot {r}_{left} \, > \, {b}_{t,right}\cdot {r}_{right}\,{{\mbox{and}}}\,\,{b}_{t,left}\cdot {r}_{left} \, > \, {r}_{sure}\hfill\\ right&{b}_{t,right}\cdot {r}_{right} \, > \, {b}_{t,left}\cdot {r}_{left}\,{{\mbox{and}}}\,\,{b}_{t,right}\cdot {r}_{right} \, > \, {r}_{sure}\\ sure&{r}_{sure}\ge {b}_{t,right}\cdot {r}_{right}\,{{\mbox{and}}}\,\,{r}_{sure}\ge {b}_{t,left}\cdot {r}_{left}\hfill\end{array}\right.$$

Because *r*_*right*_ = *r*_*left*_ = *r*_*direction*_ in our task, the above policy reduces to a comparison of the model’s confidence with the reward utility ratio *r*_*sure*_/*r*_*direction*_ between the sure-bet and correct direction choices. Since confidence increases with the absolute value of inferred coherence, ∣*μ*_*t*_∣, this reward utility ratio leads to a time-varying boundary that determines the POMDP policy as a function of inferred coherence and time in each direction (upper and lower bounds). Figure 3c shows confidence as a function of inferred coherence and elapsed time for an example POMDP model and Fig. 3d shows the model policy for an example reward utility ratio of 0.63.

With a constant observation cost, the model has up to four degrees of freedom: (i) observation cost; (ii) the true observation variance (${w}_{z}^{2}$), which shapes input samples available to the model; (iii) the learned observation variance (${\sigma }_{z}^{2}$), which the model attributes to its inputs; and (iv) the learned variance of the prior distribution (${\sigma }_{0}^{2}$). For an optimized POMDP model, however, ${\sigma }_{0}^{2}$ and ${\sigma }_{z}^{2}$ are uniquely determined by ${w}_{z}^{2}$ and observation cost. As mentioned before, ${\sigma }_{0}^{2}$ determines the prior belief, which should be consistent with the overall distribution of states and consequently, perceived observations. Moreover, ${\sigma }_{z}^{2}$ should match the model’s posterior belief with its average accuracy for each motion duration. This is possible based on the feedback given about motion direction choices (correct or wrong) after each trial (see the next section for details on estimating these parameters). Such a model, therefore, has two degrees of freedom: observation cost and ${w}_{z}^{2}$.

Note that correct posterior belief (matched with accuracy on average) is not necessary for maximizing the reward utility in choosing between the two directions because determining the sign of the sum of observations is sufficient. However, it is necessary for the wagering task where the expected reward utility of choices needs to be computed (Eq. (5)).

### Comparison of model predictions with experimental data

In our task, the stimulus viewing duration was controlled by the experimenter and subjects were required to maintain fixation throughout the duration. As a result, the cost of acquiring new observations while maintaining fixation on the stimulus could be negligible. We verified this hypothesis by comparing the model with two degrees of freedom (observation cost and *w*_*z*_) to a POMDP that uses all observations in each trial with only *w*_*z*_ as the free parameter). They were not significantly different in quality of fits even without penalizing the extra free parameter (Vuong’s closeness test^49^, *p* = 0.16 for monkey M1 and *p* = 0.07 for monkey M2; see Methods).

**Fig. 4:**
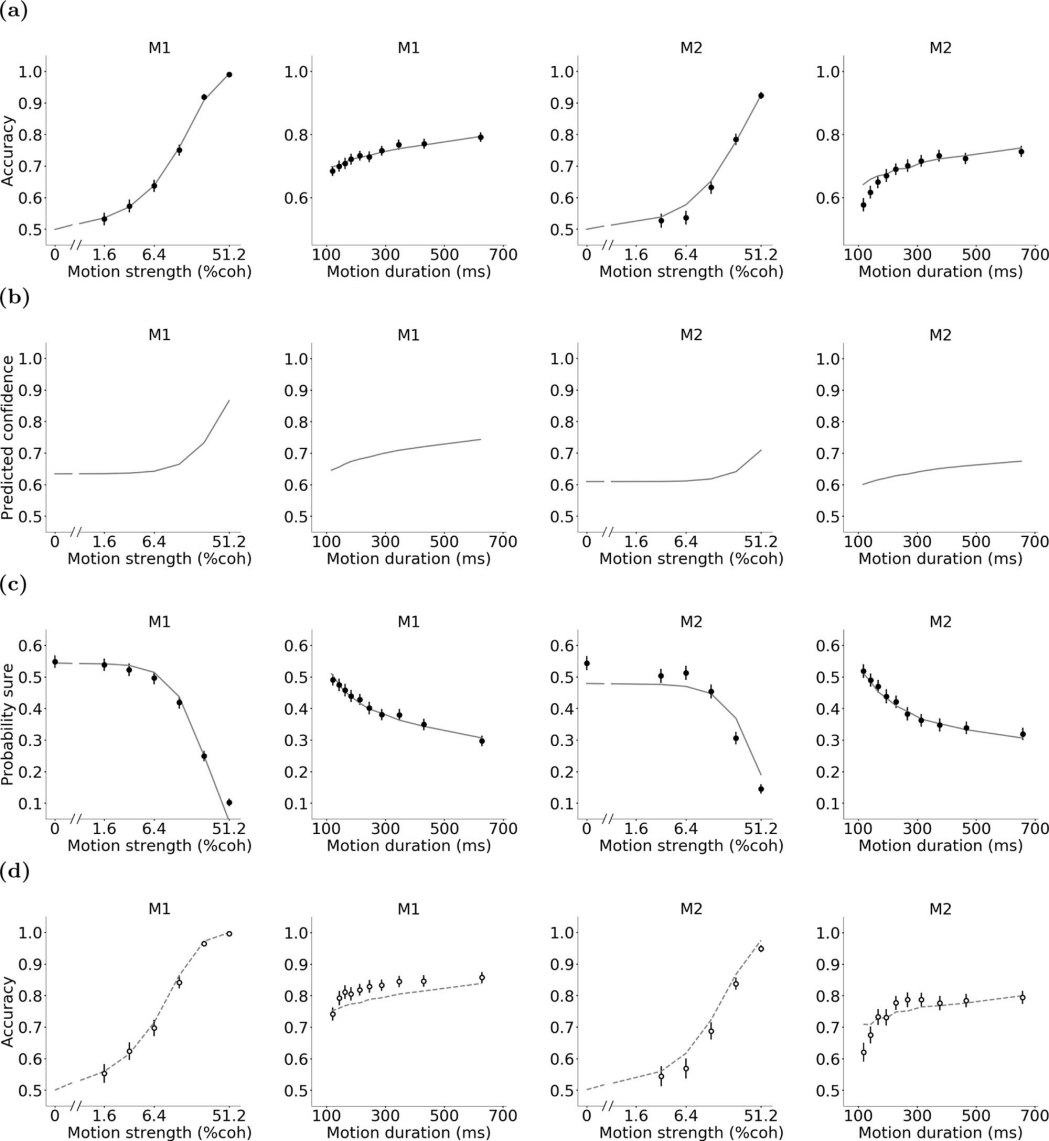
The POMDP model captures the monkeys’ behavior. **a** The model was fit to each monkey’s accuracy on trials without the sure-bet option. Solid lines are model fits and data points are the measured accuracy for each motion strength and duration for monkeys M1 and M2. **b** The model parameters obtained from the fits in (**a**) were used to predict confidence for each motion strength and duration for each monkey. **c** Predictions of the POMDP model about confidence were thresholded to fit the likelihood of choosing the sure-bet option (see Methods). **d** With the model parameters fully constrained by accuracy on trials without the sure-bet target and the likelihood of choosing the sure-bet target when it was presented to the monkey, we predicted the monkey’s accuracy on trials in which the sure-bet target was shown but ignored. Lines are model predictions. Data points are the same as in Fig. 1b, c. Error bars indicate s.e.m.

We fit the model to each monkey’s accuracy on trials in which the sure target was not shown (Fig. 4a) (*R*^2^ = 0.95 and 0.88 for monkeys 1 and 2, respectively) and obtained the observation variance ${w}_{z}^{2}$. Specifically, when there is no observation cost, the average belief about the direction right is ${{\Phi }}(\sqrt{t}c/{w}_{z})$ for trials with duration *t* and signed coherence *c*. Therefore, as we did not have access to observations in each trial, we modeled the probability of choosing the direction right with a Bernoulli distribution whose mean is ${{\Phi }}(\sqrt{t}c/{w}_{z})$ (when *c* is negative, the probability of choosing the direction right becomes less than 0.5).

Each monkey’s data were fit separately. For monkey M1, *w*_*z*_ was 0.90 while for monkey M2, it was 1.69. Based on these *w*_*z*_ values, we estimated the prior belief ${b}_{0}={{{{{{{\mathcal{N}}}}}}}}\left({\mu }_{0},\,{\sigma }_{0}\right)$ as follows: for any trial with true coherence *c* and duration *t*, we generated a sample from ${{{{{{{\mathcal{N}}}}}}}}\left(c,{w}_{z}/\sqrt{t}\right)$; the samples generated from all the trials were used to fit the Gaussian ${{{{{{{\mathcal{N}}}}}}}}\left({\mu }_{0},\,{\sigma }_{0}\right)$ via maximum likelihood estimation (MLE)^46^.

To calculate *σ*_*z*_, we fit the POMDP model’s confidence Φ(∣*μ*_*t*_∣/*σ*_*t*_) to the accuracy in all trials that the sure-bet option was not offered, using *w*_*z*_ and *σ*_0_ estimated as above. In each trial, we calculated $\mathop{\sum }\nolimits_{i = 1}^{t}{z}_{i}$, the sum of the observations generated from the actual coherence and the stimulus duration used in that trial. Using the relationship between the sum of observations, *μ*_*t*_ and *σ*_*t*_ in equation (4) we get ${{\Phi }}({\sigma }_{z}^{-2}\mathop{\sum }\nolimits_{i = 1}^{t}{z}_{i}/\sqrt{t{\sigma }_{z}^{-2}+{\sigma }_{0}^{-2}})$ as the subject’s belief about the direction right. We calculated a maximum likelihood estimate of *σ*_*z*_ by fitting this belief to the accuracy in all trials where the sure-bet option was not offered. For the fitting, the direction right choice was modeled as a Bernoulli distribution whose mean is ${{\Phi }}({\sigma }_{z}^{-2}\mathop{\sum }\nolimits_{i = 1}^{t}{z}_{i}/\sqrt{t{\sigma }_{z}^{-2}+{\sigma }_{0}^{-2}})$, where the *z*_*i*_ were sampled based on the true coherence and duration used in the trials.

One can also try to make the fit more accurate by estimating *σ*_*z*_ and *σ*_0_ iteratively. We can start with the values of *σ*_0_ and *σ*_*z*_ obtained as described above, and then readjust *σ*_0_ based on this estimated *σ*_*z*_. The readjusted *σ*_0_ can be used to fit *σ*_*z*_ again. With every such iteration, we found that the change in *σ*_0_ decreased. We repeated this process until the change in *σ*_0_ became less than our precision error. This process converged in less than 5 iterations for both monkeys. However, the readjusted *σ*_0_ values did not significantly improve the goodness of fit of the belief to the monkey’s choice. Nonetheless, we used these more accurate values in our models: *σ*_0_ was 0.46 and 0.87, and *σ*_*z*_ was 1.60 and 3.59 for monkey M1 and M2, respectively.

Finally, an important point about our model fitting process is that although the POMDP policy is deterministic, the stochasticity needed to fit the trial-by-trial choice data comes from the distribution of observations given the true stimulus.

Having estimated the model parameters based on trials without the sure-bet target, we predicted the monkey’s confidence for each motion coherence and duration (Fig. 4b). These predictions suggested a monotonic increase in confidence with motion coherence and duration, compatible with previous studies^2,4,16,50,51^.

Since the model chooses the sure-bet option when confidence (belief) is less than the reward utility ratio of the sure-bet and correct direction choice (Eq. (5)), it predicts lower probability of choosing the sure-bet target on trials with stronger motion and longer durations. Since we do not know the exact utility of reward volumes associated with the sure-bet and correct direction choices, we added a new free parameter to the model that represented the reward utility ratio and used this parameter as a threshold that the confidence was compared to on trials in which the sure-bet target was presented. Optimizing this parameter (0.63 for monkey and 0.59 for monkey 2) in order to match the predicted confidence of the POMDP model with the monkey’s behavior provided a fit with *R*^2^ = 0.90 and 0.82 for monkey M1 and monkey M2, respectively (Fig. 4c).

Since the model parameters are fully specified based on the monkey’s accuracy on trials without the sure-bet target and the probability of choosing the sure-bet target when it was presented, we could provide quantitative predictions for the monkey’s direction choice accuracy when the sure-bet target was presented but not chosen. Figure 4d shows these predictions (gray dashed lines), demonstrating that they closely match experimentally measured accuracy on trials where the monkey ignored the sure-bet option (*R*^2^ = 0.90 and 0.81 for monkey M1 and monkey M2, respectively). Trials with 0% coherence were removed from this accuracy analysis because a correct direction choice is undefined on those trials and the monkey was rewarded randomly.

As stated above, we used data from all the trials to fit our parameters (batch training of parameters). In reality, one expects the brain to estimate *σ*_*z*_ based on the history of correct and incorrect responses, with *σ*_*z*_ getting updated after each trial. We found that a trial-by-trial update method for estimating parameters based on the existing data led to results very similar to the results based on the batch approach (see supplementary materials for more details).

Because our POMDP model enables us to predict confidence from accuracy, we explored if it could also explain five well-documented discrepancies between accuracy and confidence. Based on the model’s success, we suggest that these discrepancies are neither anomalies of the decision-making process nor do they necessarily indicate a divergence of the neural mechanisms that compute choice and confidence. Rather, these phenomena are expected signatures of a decision-making process that infers the choice and its associated confidence in a unified framework.

### Hard-easy effect

The hard-easy effect, which has been documented extensively^19,27^, is the tendency to overestimate the likelihood of one’s success for difficult decisions and underestimate it for easy decisions. In the face of uncertainty about the stimulus in a given trial, the model computes confidence across all possible stimuli (marginalization). However, when the experimenter measures accuracy for each stimulus strength, this marginalization does not occur as the experimenter knows the exact stimulus on each trial^52^. The model’s uncertainty about the stimulus, therefore, causes overconfidence in difficult trials and underconfidence in easy ones.

**Fig. 5:**
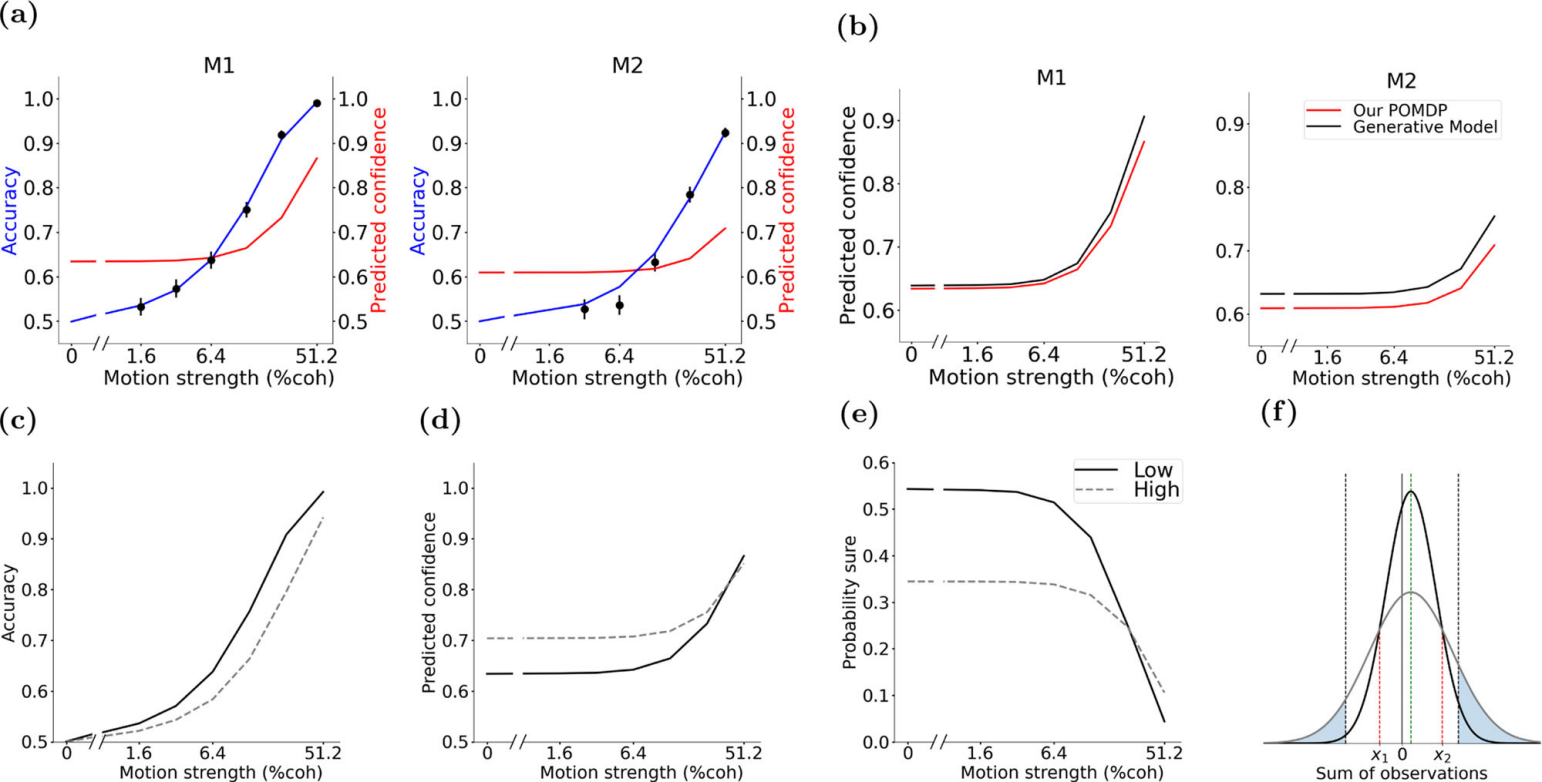
POMDP model explains the hard-easy effect and the opposing effect of a sudden change in stimulus variability on accuracy and confidence. **a** The hard-easy effect. Uncertainty about the strength of observed evidence makes the model more confident than warranted by accuracy on hard trials and less confident than warranted by accuracy on easy trials (compare blue and red curves). The psychometric and predicted confidence functions for subjects M1 and M2 are adopted from Fig. 4a, b. Error bars indicate standard error of the mean (s.e.m.). Data points are the same as in the motion strength plots in Fig. 1b. **b** While the major reason for the hard-easy effect is marginalization over coherence by the decision maker, our POMDP model also predicts a small underconfidence bias (red curve) compared to using a generative model (black curve) that assumes the exact set of coherence levels in the experiment is given. **c**–**e** Sudden increase in stimulus variability following training with lower stimulus variability reduces accuracy (**c**) but boosts confidence (**d**) and decreases the probability of sure-bet selection (**e**), or equivalently, increases the probability of sure-bet rejection. This dissociation occurs because the model relies on the observation noise learned during the lower-variability training period to render choices on the subsequent higher variability trials. **f** The increase in the probability of sure-bet rejection after a sudden increase in stimulus variability is illustrated here for two trials with the same coherence and duration but different stimulus variability. Distributions of the sum of observations for low (black Gaussian curve) and high variability (gray Gaussian curve) intersect at two points (red dotted lines). A high sure-bet rejection threshold on confidence (e.g., 85% in this example) learned during training with low variability stimuli maps to two thresholds (dotted black lines) on the sum of observations that fall outside of the intersection points. Given these fixed thresholds, the probability of sure-bet rejection is higher (larger blue filled areas under the curve) when stimulus variability is suddenly increased. This explanation is consistent with ideas presented in previous work^16,28,53^.

As shown in Fig. 5a, the POMDP model predicts this hard-easy effect after marginalization over coherence. Since the model’s Gaussian observation distribution closely approximates the true observation distribution (especially for the low coherence levels, Fig. 2b), it approximates well the confidence of the true generative model, as shown in Fig. 5b. However, the model does exhibit a small underconfidence bias since it considers the full range of continuous coherence levels. As expected, this bias is larger in the region where the coherence levels are further apart (and consequently the observations overlap less), which in our task are the easier trials (higher coherences; see monkey M1’s plot), and for experiments with Monkey M2 where the task did not use the 1.6% coherence level. Overall, these results illustrate how differences between the real world model and the decision maker’s internal model (in our case, discrete versus continuous distribution for coherence; Fig. 2a) could create a bias in confidence for an optimal decision maker.

### Opposing effects of the variability of observations on choice and confidence

A common observation in past studies has been that increasing the variability of the stimulus reduces subjects’ accuracy but increases their confidence about their choices^16,28,53^. Our POMDP model shows that this seemingly paradoxical effect of stimulus variability arises naturally in an optimal inference framework when the subject does not have access to the true model of the environment (in this case, the true observation noise).

Stimulus variability effects have been explored in tasks where subjects were trained using a baseline (lower) stimulus variability, before being tested on higher variability. Further, trials with different levels of stimulus variability were randomly intermixed. Consequently, our model postulates that subjects continued to rely on the observation noise learned during initial training, and used this model for choice and confidence in the high variability trials. Higher variability (larger ${w}_{z}^{2}$) decreases accuracy (Fig. 5c, left plot) by generating more overlapping observations for different motion directions.

Higher variability also generates extreme observations (far from the mean) more often, including ones in favor of the incorrect choice (e.g., negative coherence observations when the true coherence is positive). These extreme observations, although frequent in the high variability regime, are not expected based on the observation noise learned during training in a low variability regime. As a result, the POMDP model considers these extreme observations highly discriminative, resulting in a higher confidence with a concomitant decrease in the probability of choosing the sure-bet option when presented^16^, especially in high and medium difficulty level trials (Fig. 5d, e).

To further understand this phenomenon, we adopted the intuitions and ideas suggested in previous work^16,28,53^. We explored the change in probability of rejecting the sure-bet option (indicating high confidence) in trials with a low coherence level *c* for a specific stimulus duration *t* and no observation cost. Suppose the true coherence is positive (the case where the coherence is negative is similar). The sum of observations comes from a Gaussian distribution with mean *tc* and variance $t{w}_{z}^{2}$. Choosing or rejecting the sure-bet option can be mapped to two thresholds on the sum of observations, one for each direction. This mapping depends on *σ*_0_ and *σ*_*z*_, and consequently *w*_*z*_ (indirectly).

Figure 5f shows the distribution of the sum of observations for two example stimuli with the same positive coherence level (+6.4%, green dotted line) and duration (250 ms) but different variability, with low variability shown as the black Gaussian curve and high variability as the gray Gaussian curve. The plot also shows the sure-bet selection/rejection thresholds (black dotted lines) learned during training with the low variability curve for this example with +6.4% coherence. The low and high variability curves intersect each other at two points (red dotted lines). Note that the sure-bet selection/rejection thresholds (black dotted lines, fixed after training) are lesser than or greater than the intersection points (red dotted lines), implying that these learned thresholds are in the area where probability density for the higher variability stimulus (gray curve) is higher. This means that the area under the curve beyond these thresholds (blue filled areas), equal to the probability of sure-bet rejection (indicating high confidence), is larger for the high variability stimulus than the low variability stimulus (narrower dark curve) used during training. These results illustrate how higher confidence can be generated when the stimulus becomes more noisy.

### Discrepancy of sensitivity for accuracy (${d}^{\prime}$) and confidence (meta-${d}^{\prime}$)

**Fig. 6:**
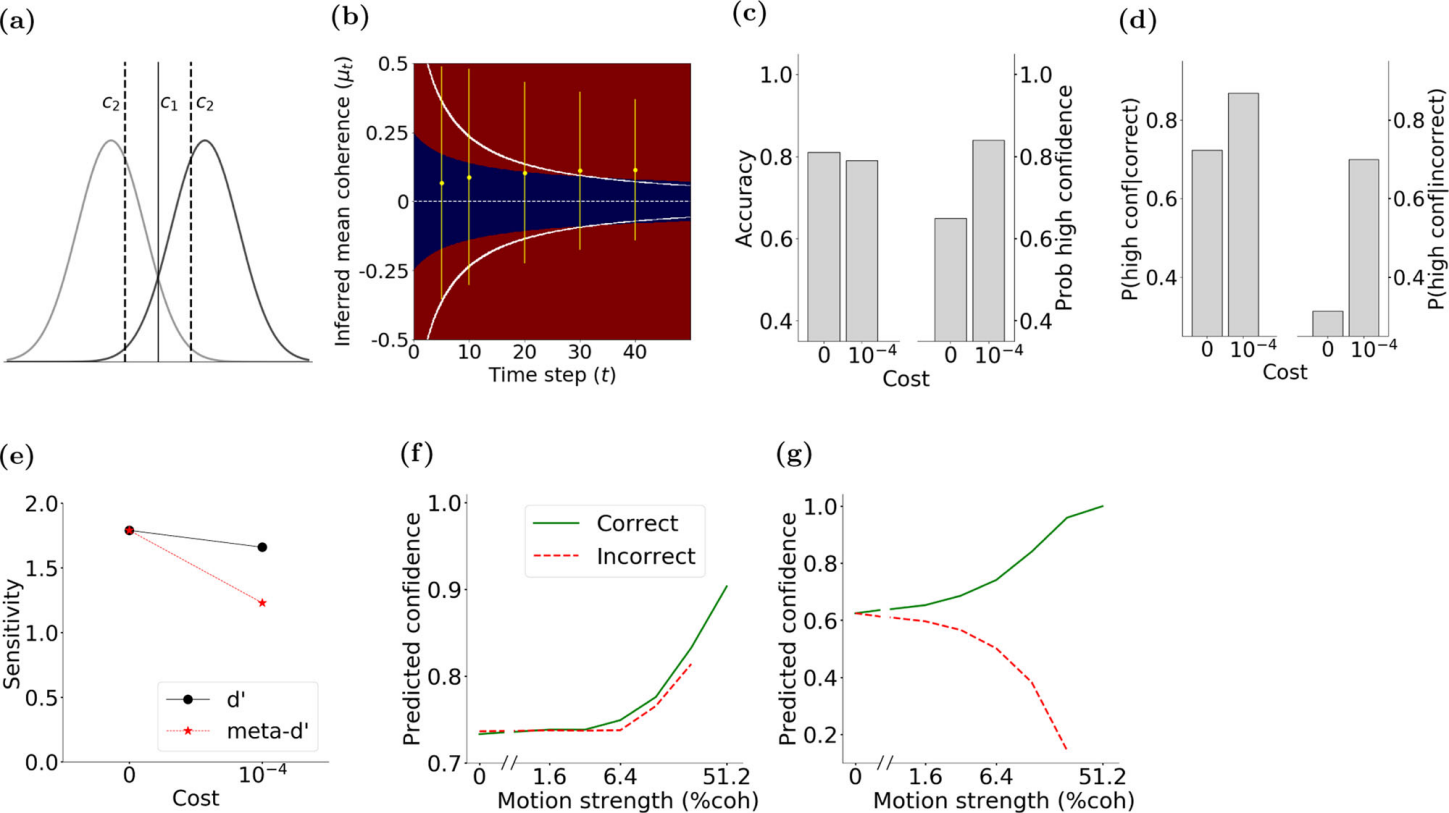
POMDP model explains different values for ${d}^{\prime}$ and meta-${d}^{\prime}$, and different patterns of confidence in reaction-time experiments. **a** A signal detection theory framework predicts identical ${d}^{\prime}$ and meta-${d}^{\prime}$ when the same observations inform both choice and confidence rating. The competing stimuli (e.g., right and leftward motion of a particular coherence) give rise to two observation distributions. The *c*_1_ criterion is used for choosing right and left, and the *c*_2_ criteria are used to report low or high confidence for each choice. ${d}^{\prime}$ quantifies type 1 sensitivity: the distance between the distributions in units of standard deviation. Meta-${d}^{\prime}$ quantifies type 2 sensitivity: the separation of the two distributions compatible with hit rate and false alarm rate of confidence reports: *p* (high conf∣correct) and $p\left(\,{{\mbox{high conf}}}| {{\mbox{incorrect}}}\,\right)$, respectively. In SDT, ${d}^{\prime}$ and *c*_1_ fully constrain meta-${d}^{\prime}$ and an optimal meta-cognitive observer must have equal ${d}^{\prime}$ and meta-${d}^{\prime}$^30^. **b** Observation noise could cause highly variable *μ*_*t*_ at the beginning of a trial, and thus temporarily produce excessive confidence. This excessive confidence may become permanent if the decision-making process is stopped by reaching the termination bounds. Solid white lines show the two decision termination bounds (observation cost, 10^−4^). Thresholds for separating low and high confidence ratings are shown as boundaries between blue (low confidence) and red (high confidence) regions. The horizontal dashed line shows the boundary that separates right and left direction choices based on the sign of the inferred coherence. Yellow dots and lines show mean ± 2 × *s*. *d*. (95% of the distribution mass) of the inferred coherence for a particular stimulus strength (c = +12.8%) at a few different time steps (10 ms per step). Temporary excessive confidence due to early termination is more prominent for the incorrect trials (negative *μ*_*t*_ in this simulation). **c** Early termination can cause a modest reduction of accuracy and a marked increase of high-confidence ratings. **d** Early termination can cause a larger increase in the probability of high confidence ratings for incorrect than correct choices. **e** Changes in accuracy (**c**) and confidence ratings (**d**) can lead to a larger drop in meta-${d}^{\prime}$ than ${d}^{\prime}$. Model parameters are identical to those for monkey M1, except for the observation cost. **f** For experiments with simultaneous reports of choice and confidence, our model predicts higher confidence for incorrect choices on trials with stronger stimuli (red dashed line). This pattern is partly caused by lower decision times for stronger stimuli and the dependence of model confidence on elapsed time (Fig. 3c). **g** In contrast, for sequential reports of choice and confidence, our model predicts reduced confidence for incorrect choices for stronger stimuli (red dashed line). This is due to sensory and motor delays that render the last observations inconsequential for the choice but the model uses those observations to refine its confidence report following the choice.

The POMDP model also explains experimentally observed differences between the sensitivity of accuracy and confidence to observations, commonly quantified with ${d}^{\prime}$ and meta-${d}^{\prime}$, respectively^30^. ${d}^{\prime}$ and meta-${d}^{\prime}$ are defined based on a signal detection theory (SDT) framework. ${d}^{\prime}$ quantifies the difference of sensory evidence distributions underlying the probability of correct and incorrect choices while meta-${d}^{\prime}$ is related to the distribution of confidence ratings for those choices. For a binary confidence rating (low or high confidence, similar to rejecting or choosing the sure-bet option), meta-${d}^{\prime}$ contrasts the probability of a high confidence rating for a correct response with that of an error. Some studies have reported that confidence ratings are not consistent with the sensitivity of the choice accuracy (${d}^{\prime}$)^30,54–56^. However, for an SDT ideal observer meta-${d}^{\prime}$ and ${d}^{\prime}$ have to be similar in the absence of variability in the confidence rating threshold (Fig. 6a). Therefore, it has been suggested that the different meta-${d}^{\prime}$ and ${d}^{\prime}$ in experimental data must be due to loss of information for confidence judgments or different neural mechanisms for confidence and choice^30,31^.

In the absence of an observation cost, where the POMDP model uses all available evidence, its ${d}^{\prime}$ and meta-${d}^{\prime}$ match each other, similar to SDT. That would be true regardless of whether the decision maker does or does not have access to the exact model of the environment. However, if there is an early termination of information gathering, then meta-${d}^{\prime}$ could diverge from ${d}^{\prime}$. This discrepancy emerges in the model not because of distinct mechanisms for choice and confidence, but because early terminations of the decision-making process have quantitatively distinct effects on the choice accuracy and the likelihood of high confidence ratings for correct and incorrect choices. Because early terminations curtail the use of evidence, they reduce accuracy and, therefore, decrease ${d}^{\prime}$. Further, in the face of uncertainty about the reliability of evidence, early terminations are associated with higher confidence (Figs. 3c and 6b). This combination means that for a wide range of model parameter values, the model makes more errors but it is also more confident about its choices compared to a model without an observation cost. Critically, the confidence is inflated more on error than correct trials (Fig. 6d, reducing meta-${d}^{\prime}$. This reduction could be substantially larger than the reduction of ${d}^{\prime}$. Consequently, the model could generate meta-${d}^{\prime}$ values smaller than its ${d}^{\prime}$, even though it computes the choice and confidence through the same optimal process.

Figure 6 illustrates these effects by simulating intermediate coherence (+12.8%) trials with 400 ms duration and subjecting the model choices and confidence to the ${d}^{\prime}$ and meta-${d}^{\prime}$ calculations. Model parameters are inherited from Monkey M1 except for the addition of an observation cost (10^−4^/observation). Early in the trial, observation noise can temporarily produce large positive or negative inferred *μ*_*t*_, and thus high confidence (Fig. 6b, yellow lines illustrate *mean* ± 2 × *s*. *d*. of the inferred *μ*_*t*_). Such large *μ*_*t*_ are much less likely at later times because of the correction of excessive early confidence with additional observations. These later corrections, however, are prevented if the termination bounds (Fig. 6b, white lines) are reached earlier. Such occasional early terminations reduce the model accuracy by only 2% for this motion coherence (from 81% with no observation cost to 79%), but increase the overall probability of high confidence choices by 19% (from 65 to 84%) (Fig. 6c). The corrective effect of additional observations on confidence is more pronounced when the initial choice is incorrect as new observations are more likely to cancel the extreme noise that lead to early error choices. Consequently, early terminations increase the fraction of high confidence responses for incorrect choices by 39% (from 31 to 70%), whereas the increase for correct choices is 15% (from 72 to 87%) (Fig. 6d). This reduces the contrast of confidence for correct and error choices, resulting in a reduction of meta-${d}^{\prime}$. This reduction is larger than the very modest reduction of ${d}^{\prime}$, bringing the ratio of meta-${d}^{\prime}$ to ${d}^{\prime}$ to 0.74, significantly below 1 (Fig. 6e).

The reduction of meta-${d}^{\prime}$ could happen even when the overall confidence rating does not increase in the model, as meta-${d}^{\prime}$ depends on the contrast of confidence for correct and error choices, which could be differentially affected by early terminations with or without an overall confidence increase. Generally, meta-${d}^{\prime}$ to ${d}^{\prime}$ ratios below 1 are common for a wide range of POMDP model parameters matching a common result in past behavioral studies^31^. Further, the model predicts a mismatch between meta-${d}^{\prime}$ and ${d}^{\prime}$ in reaction-time tasks, where the decision maker initiates a response as soon as reaching a decision. Overall, distinct ${d}^{\prime}$ and meta-${d}^{\prime}$ values can arise in the POMDP framework not because different information shapes choice and confidence, but rather when the decision-making process can stop due to a termination criterion without utilizing all the available information. This important alternative explanation has been largely neglected in past explanations of mismatched meta-${d}^{\prime}$ and ${d}^{\prime}$.

### Sensitivity of confidence measurements to simultaneous versus sequential reports of choice and confidence

The POMDP model can also be applied to reaction-time tasks (besides fixed-duration tasks), where subjects report their choice as soon as they are ready. In these tasks, the experimenter may ask for either a simultaneous or sequential report of choice and confidence^12,27,29^. Past experiments have shown that for simultaneous reports of choice and confidence, confidence for incorrect choices often increases with stimulus strength, compatible with the predictions of bounded accumulation models such as the DDM^12,29^. However, for sequential reports, confidence for incorrect choices decreases with stimulus strength, compatible with the predictions of signal detection theory^11,27,29^.

The POMDP model predicts both patterns (Fig. 6f, g). Consider first the case of simultaneous report of confidence and choice. As previously shown in Fig. 3c, a decision to stop gathering more observations after a short period of time is associated with higher confidence. Fig. 6b shows an example where observation noise may cause the decision bound to be reached early in the trial leading to a confident but incorrect decision (a large negative inferred mean coherence when the true coherence is positive). When coherence is high, incorrect decisions after many observations are unlikely. However, as in our example, early extreme observations may cause early termination and incorrect confident choices in high coherence trials. As a result, incorrect high coherence trials will have much shorter duration and therefore higher confidence compared to low coherence trials (Fig. 6f).

To explain the confidence pattern in sequential report of confidence and choice, consider the difference between fixed-duration and reaction-time tasks. In a fixed-duration task, the subject may commit to a choice early in the trial if the cost of gathering observations is higher than the increase in the expected utility from gathering more observations. However, the trial does not terminate with early commitment to a decision and the subject has to wait till the end of the trial to obtain the reward. In a reaction-time task, however, the subject controls the length of the trial and has a greater incentive to commit to a choice early to get the reward (assuming the choice is correct)^57^, in addition to minimizing the overall cost of gathering observations. Furthermore, making faster decisions means more quickly moving to the next trial (assuming a time penalty for an incorrect choice did not occur) with the potential for more reward.

On the other hand, in a reaction-time task, even after selecting the choice, observations may continue to be gathered if presented by the experimenter. The sensory and motor delays in the neural circuits underlying decisions usually amount to around 250 ms or more, leading to the availability of these extra observations to the decision maker. These observations do not contribute to the choice and they do not contribute to the confidence report when it is simultaneous with the choice report. However, sequential report of choice and confidence opens up the possibility of revising confidence based on these last few post-choice sensory observations.

Confidence in incorrect trials is especially susceptible to such revisions. In easy (i.e., high coherence) trials, when early extreme observations may have led to an incorrect choice (e.g., Fig. 6b), the post-choice observations are very likely to be in favor of the correct choice, causing the subject to lower their confidence after making a decision. In fact, since easy trials with incorrect choices are typically very short, the post-choice observations might even lead to a change of mind by the subject^29,58^, and consequently decreasing confidence (lesser than 0.5 in some cases) as a function of increasing stimulus strength (coherence) in incorrect trials (Fig. 6g).

### Effects of choice-congruent and choice-incongruent evidence

The last phenomenon we explore in this section is whether confidence reports are more strongly influenced by evidence congruent with the choice compared to incongruent evidence. Previous studies have reported that whereas choice is shaped by the balance of evidence for different options, confidence is more strongly shaped by choice-congruent evidence^15,20^. These results have been interpreted as support for processes that compute confidence after the choice by readjusting the weight of evidence based on the choice (a form of confirmation bias). Our POMDP model demonstrates that this interpretation is not unique. Rather, existing experimental results could be explained without assuming distinct choice and confidence processes, or choice-dependent re-weighting of evidence.

A key feature of analyzing data based on the POMDP framework is to distinguish the observations used by the subject and those analyzed by an experimenter who monitors the subject’s behavior. Because the experimenter does not have access to the subject’s observations as encoded in the nervous system, analysis of data has to rely on the expected distribution of evidence given stimulus properties (e.g., using filters on the stimulus^20^) or recordings from the brain (e.g., electrocorticography or ECoG^15^). Such estimated observations could markedly diverge from the actual observations used by the subject. A wide variety of mechanisms could underlie such a divergence, including decision bounds or other termination criteria unknown to the experimenter, sampling rates that mismatch the stimulus design, shifts in spatial or temporal attention during a trial, noise in the representation of sensory information by neural responses, or recording noise from the brain.

To clarify the significance of the divergence of observations used by the decision maker and those the experimenter uses to investigate behavior, consider the case where a decision maker uses only a proportion of the observations analyzed by the experimenter (*n* out of the total *t* samples, *n* < *t*). In this situation, the *t* − *n* samples not used by the subject act as noise in the analyses. Classification of choice based on stimulus fluctuations reveals equal and opposing influence of stimuli supporting different alternatives as both used and unused observations come from the same distribution. However, conditional on the subject’s choice, the proportion of choice-congruent observations is higher in the portion of the stimulus used by the subject, compared to the unused portion. This is simply because the sum of random variables drawn independently from the same distribution being positive is evidence in favor of each of these variables being positive. If we reorder the observations in a way that *z*_1_,…,*z*_*n*_ become the ones used by the subject and *z*_*n*+1_,…,*z*_*t*_ are the unused ones (only to simplify the equations), we have:

6 $$\mathop{\sum }\limits_{j = 1}^{n}{z}_{j} \, > \, 0\to \forall \,1\le i\le n\,\& \,n+1\le l\le t:P({z}_{i} \, > \, 0) \, > \, P({z}_{l} \, > \, 0)$$

And also:

7 $$\mathop{\sum }\limits_{j=1}^{n}{z}_{j} \, > \, 0\to \frac{1}{n}{\mathbb{E}}\left[\mathop{\sum }\limits_{j=1}^{n}{z}_{j}\right] \, > \, \frac{1}{t-n}{\mathbb{E}}\left[\mathop{\sum }\limits_{j=n+1}^{t}{z}_{j}\right]$$

The inequalities of Eqs. (6) and (7) have a profound side effect for quantification of the influence of individual observations on confidence. If we divide observations based on whether they support the choice, the ratio of total choice-congruent observations to total incongruent observations will be higher for the set of observations used by the decision maker (the *n* samples) than those used in the experimenter’s analyses (all *t* samples). As a result, a classifier that uses all *t* observations to predict the decision maker’s confidence has to give a larger weight to the choice-congruent observations to compensate for the dilution of congruent evidence caused by the unused stimulus samples.

**Fig. 7:**
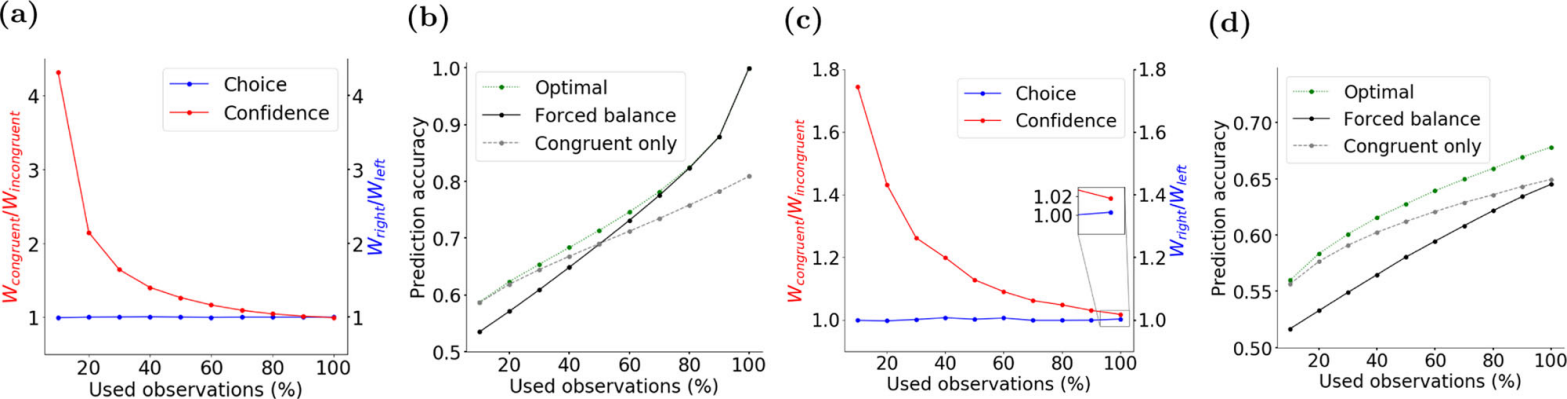
POMDP model explains seemingly higher influence of choice-congruent evidence on confidence ratings. Discrepancy in the observations used by a decision maker and those used by an experimenter studying the decision maker’s behavior could lead to biased interpretation of experimental results. **a** We simulated a POMDP model that uses a fraction of observations available in a trial unbeknownst to the experimenter. The observations supporting opposing choices equally inform the model’s behavior. However, an experimenter who uses a classifier to predict choices and confidence based on all observations in the trial finds an apparently larger influence of choice-congruent observations on confidence. **b** Forcing the classifier to have balanced weights for all observations causes lower prediction accuracy of confidence ratings, especially when the proportion of used evidence is low. In such cases, even a model that totally ignores choice-incongruent observations performs better than the balanced model. However, the better performance of models with imbalanced weights does not reflect the decision making process. It stems merely from the experimenter’s lack of knowledge about the observations used by the simulated model. **c**, **d** Same as (**a**, **b**) but observations accessible to the experimenter are noisy estimates of observations available to the decision maker. Such noise reduces the prediction accuracy of the experimenter’s classifier, but more importantly, it also causes imbalanced weights in the optimal classifier (**c**) and lower performance of the balanced classifier (**d**). That is true even when both the decision maker and experimenter use all the available observations (see inset box in (**c**)). The noise in these simulations comes from a zero-mean Gaussian distribution with a variance 25% larger than ${w}_{z}^{2}$.

To demonstrate this, we simulated a fixed-duration version of the random dots task with binary confidence ratings (low vs. high). For any stimulus strength and with *n* < *t*, a logistic classifier fit to the proportion of high confidence ratings by the POMDP model yielded larger weights for congruent than incongruent observations (Fig. 7a). In contrast, a logistic classifier fit to right and left choices based on stimulus fluctuations revealed equal and opposing weights for positive and negative samples as both used and unused observations come from the same distribution. As expected from Eq. (7), the imbalance of the weights of the confidence was more pronounced for smaller *n*. To further demonstrate the inevitable imbalance of the weights, we compared the prediction accuracy of the confidence classifier with two-alternative classifiers: one forced to have balanced weights for congruent and incongruent observations and a second classifier that had access only to the congruent evidence (Fig. 7b). Similar comparisons were used in past studies^15^. The confidence classifier with balanced weights had a lower prediction accuracy, especially for low *n*, where its accuracy was even lower than the classifier that totally ignored incongruent observations.

With similar reasoning, choice-congruent observations gain a higher weight in predicting confidence when the experimenter uses a subset of the observations used by the subject (Supplementary Fig. 2).

Although the example above focuses on a particular source of discrepancy between the observations used by the decision maker and experimenter (different number of used samples), the conclusion generalizes to other sources of discrepancy. Some of these sources such as neural noise are almost always present and quite difficult to correct the analyses for. Essentially, the observations analyzed by the experimenter are almost always noisy estimates of the observation used by the decision maker: ${z}_{j}^{experimenter}={z}_{j}^{subject}+\zeta$, where *ζ* denotes noise with an often unknown magnitude. The neural noise causes the same dilution of choice-congruent evidence explained in the example above. Consequently, the experimenter is bound to estimate a higher weight for congruent samples in the analyses even when *n* = *t* (Fig. 7c) and even though such weight imbalance may not exist for the decision maker. Large enough noise can even make a classifier that only uses choice-congruent observations better than a balanced classifier (Fig. 7d).

### Relationship between POMDP and drift diffusion models

**Fig. 8:**
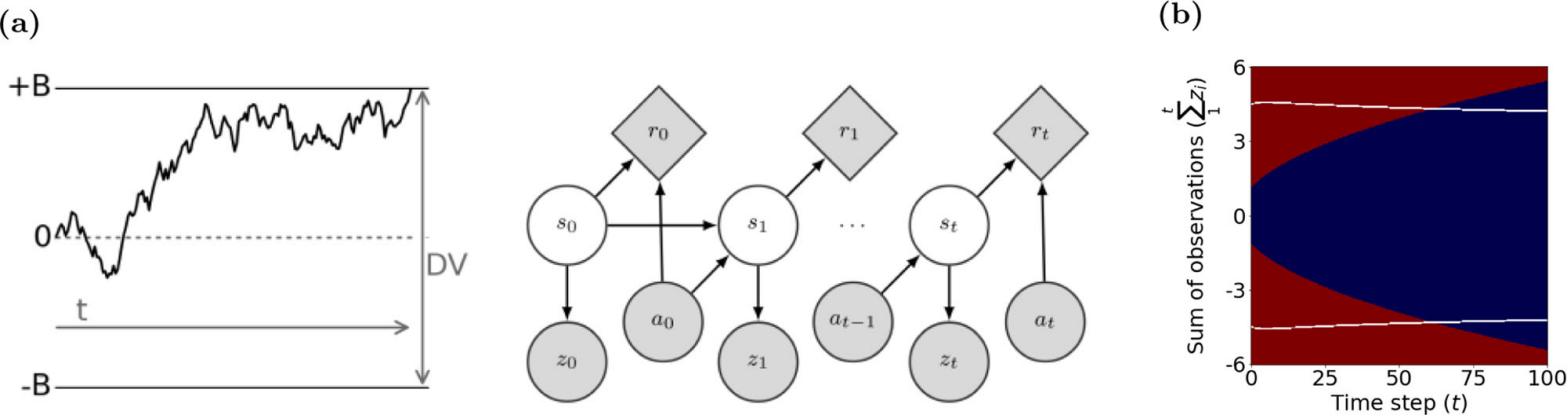
The POMDP policy can be implemented by a drift diffusion model (DDM) with collapsing bounds. **a** (Left panel) In the standard DDM, the decision variable (DV) is the sum of observations over time. The process stops when the DV reaches one of the static decision bounds (+B or −B). (Right panel) Graphical model for a POMDP. For each time step (indicated by the subscripts 0, 1, ..., *t* − 1, *t*), *r* is the reward gained due to action *a* in hidden state *s*. *z* is the observation in hidden state *s*. The POMDP model infers a posterior probability distribution over hidden states at each time step based on past observations and actions. In the motion discrimination task, the actions are committing to a choice or making another observation. The model commits to a choice when the expected increase in the probability of a correct response is not worth the cost of an extra observation. **b** The time-varying bounds on *μ*_*t*_ in the POMDP policy map (e.g., solid white lines in Fig. 6b) have equivalent time-varying bounds on the DV in the DDM (Eq. (10); white lines in this panel). Similarly, the low and high confidence regions (blue and red regions respectively) of the POMDP policy map in Fig. 6b have equivalents in the DDM as shown here.

A simple mathematical model that has been extensively used to provide quantitative fits to behavior and explain neural activity in various brain regions is the drift diffusion model (DDM)^32^. DDM assumes that each observation confers evidence in favor of one choice and an equal amount of evidence against the other choice (Fig. 8a). Integration of sensory evidence over time provides a decision variable (DV) that tracks the total evidence in favor of each choice. In most formulations of DDM, two bounds above and below the initial value of the DV (+B and −B in Fig. 8a) act as termination criteria for the decision. As soon as the DV reaches one of these bounds, the decision-making process stops and the choice associated with the bound is made. In cases where the stimulus terminates before a bound is reached, the choice with the most supporting evidence is selected^45^. For the direction discrimination task, the decision variable, *V*_*t*_, is updated with each new sensory observation according to:

8 $${V}_{t}={V}_{t-1}+{z}_{t}$$

where *V*_*t*−1_ is the DV at time *t* − 1 and *z*_*t*_ represents the momentary sensory observation drawn from a Gaussian distribution with mean *c* and variance ${w}_{z}^{2}$. *V*_0_ is initialized to zero when the prior probability and expected reward of the two choices are equal. Therefore, prior to reaching a bound, *V*_*t*_ equals the sum of observations $\mathop{\sum }\nolimits_{j = 1}^{t}{z}_{j}$ at time *t*.

The DDM as described above has previously been linked to probabilistic reasoning between two categories as in signal detection theory^1,59^. These previous models explain choice accuracy but the subject’s belief when there are multiple motion coherence levels was not addressed. A later model by Drugowitsch et al.^43^ addressed this issue by adding Bayesian reasoning on the drift rate of the DDM but the generative model was assumed to be known and exact.

Our POMDP model allows for both a learned generative model and a belief update rule that can be mapped to the DDM. Taking the ratio of the two update rules in Eq. (4), we obtain:

9 $$\frac{{\mu }_{t}}{{\sigma }_{t}^{2}}={\sigma }_{z}^{-2}\mathop{\sum }\limits_{j=1}^{t}{z}_{j}={\sigma }_{z}^{-2}{V}_{t}$$

where the second equality is based on the definition of the DV in DDM (Eq. (8)). Thus, the Bayes update of the inferred coherence, *μ*_*t*_, can be achieved via addition in the DDM. This means that there is a unique mapping from *μ*_*t*_ and ${\sigma }_{t}^{2}$ of a POMDP model to the *V*_*t*_ and *t* of the DDM.

This mapping holds in the presence of a bound in the DDM^6,60^. Moreover, the termination criterion in the POMDP model translates to a unique bound in the DDM. As shown in Figs. 3b and 6b, the policy in the POMDP model can be expressed as a bound in the space defined by inferred mean coherence, *μ*_*t*_, over time. This bound on *μ*_*t*_ has an equivalent bound on *V*_*t*_ in the DDM. In general, if ${{{\Theta }}}^{\prime}\left(t\right)$ is the time-varying termination criterion applied to *μ*_*t*_ in a POMDP model (as in Fig. 3b), the equivalent bound, ${{\Theta }}\left(t\right)$, on *V*_*t*_ in the DDM is given by:

10 $${{\Theta }}\left(t\right)=\frac{{{{\Theta }}}^{\prime}\left(t\right)}{{\sigma }_{z}^{-2}{\sigma }_{t}^{2}}=\left(t+\frac{{\sigma }_{z}^{2}}{{\sigma }_{0}^{2}}\right){{{\Theta }}}^{\prime}\left(t\right)$$

where the first equality derives from Eq. (9) and the second from Eq. (4). Similarly, confidence ratings can be expressed as time-varying boundaries in the DDM. Figure 8b shows the decision bound and confidence rating boundaries based on the accumulated evidence in the DDM derived to match the POMDP model in Fig. 6b.

Overall, both the inference process and the termination criterion of the POMDP model can be implemented with a DDM, suggesting that the neural circuitry for integration of sensory evidence could effectively be implementing the POMDP policy explained in this paper.

## Discussion

We present a Bayesian framework based on POMDPs that accounts for choice and confidence in perceptual decision-making tasks. Our framework explains the effects of observation cost and task structure on choice and confidence. It also elucidates how the observation noise learned by a Bayesian decision maker may systematically differ from the veridical observation noise, and how this difference influences prior beliefs and confidence. We use our framework to explain the emergence of commonly observed discrepancies between confidence and choice accuracy. Further, we show how our POMDP model can be mapped to bounded evidence accumulation models^4,32,61^ and potentially be implemented by the same cortical and sub-cortical neural networks implicated in the decision-making process^62,63^.

We tested our model using the behavioral data of monkeys performing a direction discrimination task with post-decision wagering^2^. The monkeys’ choice accuracy provided quantitative predictions about subjective confidence. These predictions fit the monkey’s opt-out behavior in our task, indicating that the monkey’s confidence matches the POMDP framework. Prediction of confidence purely based on choice accuracy is a remarkable feat for a computational framework, especially considering systematic discrepancies between the two^18^.

Discrepancies between accuracy and confidence have been commonly considered as evidence for suboptimal decision-making or distinct processes that underlie choice and confidence. Our POMDP framework challenges these interpretations by showing that a normative Bayesian decision maker optimizing a reward function elicits the same discrepancies between confidence and accuracy as those identified in humans and experimental animals. We explored five common discrepancies in this paper. Two of them arise from the decision maker’s incomplete knowledge of the environment. The first one is the hard-easy effect, where decision makers are over-confident for difficult choices and under-confident for easy choices^6,27^. This effect arises from the model marginalizing over the unknown stimulus strengths and the model’s approximation of a discrete set of stimulus strengths by a Gaussian model. The second discrepancy is the opposing effects of stimulus variability on choice and confidence, where subjects become less accurate but paradoxically over-confident about more variable stimuli^16^. This effect arises from another form of incomplete knowledge about the environment: attribution of the observation noise learned in environments with low variability to newly experienced conditions with higher variability.

The other three discrepancies between accuracy and confidence are explained by our model as arising from the experimenter’s incomplete knowledge of the subjects’ decision-making process. The third discrepancy is the inequality of ${d}^{\prime}$ and meta-${d}^{\prime}$, which has attracted much attention lately as experimental support for distinct processes underlying choice and confidence^30^. We show that this difference could arise even when a unitary process shapes both choice and confidence, as in our model. A cost-based termination criterion for the decision-making process could affect accuracy and confidence differently. Whereas the overall accuracy decreases due to early termination, confidence can increase especially for incorrect choices, causing unequal ${d}^{\prime}$ and meta- ${d}^{\prime}$. It is therefore impossible to uniquely interpret meta-${d}^{\prime}$ in the absence of accurate knowledge about the form of the termination criterion. However, common task designs for measuring choice and confidence often preclude such knowledge.

It is also important to mention that in the presence of observation cost, meta-${d}^{\prime}$ depends on the confidence rating threshold in the POMDP model. This sensitivity questions one of the key assumptions in the definition of meta-${d}^{\prime}$—independence of meta-${d}^{\prime}$ from the confidence rating criterion—and cautions against interpretations of meta-${d}^{\prime}$ results without knowing the variability of confidence rating thresholds across subjects in an experiment.

The fourth discrepancy is based on the observation that confidence reports differ in experiments that interrogate confidence simultaneously with the choice^12^ or after the choice^29^. This difference arises in our model because sequential reports of choice and confidence allow revising one based on information unused for the other. For example, when confidence reports follow the choice, sensory observations in the processing pipeline that were unavailable at the time of the choice could change confidence^29^.

The fifth discrepancy that the model explains is the hypothesis that confidence is more strongly influenced by choice-congruent observations than choice-incongruent observations^15,20^. Although these experimental results could indicate post-choice re-weighting of observations for calculation of confidence, they could also arise from the experimenter’s incomplete or inaccurate knowledge of the exact observations used by the decision maker. Many factors could engender such inaccuracy, including neural noise, which is often inaccessible to the experimenter, device noise, which is difficult to eliminate for electrophysiological and imaging techniques, or termination criteria for the decision-making process, which the experimenter may be unaware of or unable to identify.

To clarify our conclusion, we do not imply that dual or hierarchical processes for choice and confidence could not exist. Nor do we exclude the possible existence of mechanisms that revise confidence by post hoc choice-dependent re-weighting of the observations. Rather, we conclude that existing experimental results are insufficient to support such mechanisms as they are also compatible with simpler, more parsimonious mechanisms in which a unitary process underlies both choice and confidence. It is further illuminating that the unitary process explored in this paper is based on POMDPs, a normative Bayesian framework based on expected reward maximization. In light of our POMDP model, existing experimental results should be carefully reconsidered and better experiments should be developed to test the necessity of more complex or disparate mechanisms for choice and confidence.

We applied the POMDP framework to a fixed-duration task where the stimulus duration was controlled by the experimenter. The framework can also be used to model animal and human behavior in reaction-time tasks^24^ (as in Section “Sensitivity of confidence measurements to simultaneous versus sequential reports of choice and confidence”). In fact, reaction-time tasks might offer better opportunities to study choice and confidence. When analyzing fixed-duration tasks, long stimulus durations are problematic because a multitude of mechanisms, including decision bounds, time-varying attention, or task engagement, could cause partial use of sensory information unbeknownst to the experimenter. Short stimulus durations are not immune to misinterpretations either. Short stimuli can cause neural responses that last longer than the stimulus duration^64–66^, providing an opportunity for selective sampling. Moreover, short-term mnemonic mechanisms and active revision of choice and confidence provide additional opportunities for dissociating the observations used by the decision-making process from those assumed by the experimenter. Reaction-time task designs where subjects control the stimulus viewing duration, combined with monitoring and manipulation of neural responses in sensory and decision-making circuits, would improve experimental control and enable more accurate interpretation of experimental results^67^.

We conclude by noting that simple bounds on decision variables, as employed in traditional models of decision making, might not be sufficient to capture the types of complex policies (mappings of beliefs to actions) required in dynamic environments and in tasks more complex than the random dots task. In such cases, the POMDP model offers a powerful and flexible framework for decision making as it allows (i) arbitrary probability distributions for the prior and observation functions, (ii) arbitrary state transition functions conditioned on the decision maker’s actions, and (iii) policies that are not restricted to bounds on decision variables and that implement arbitrary mappings of beliefs to actions^24,25,68,69^. Testing these more general attributes of the POMDP model in animal and human experiments remains an important direction for future research.

## Methods

### Direction discrimination task with post-decision wagering

Complete details of our decision making task involving two macaque monkeys (M1 and M2) are provided in a previous publication^2^. All training and data collection procedures conformed to the National Institutes of Health Guide for the Care and Use of Laboratory Animals and were approved by the University of Washington Animal Care Committee. The two monkeys were trained to report the net direction of motion of a stimulus of randomly moving dots, a fraction of which moved in a particular direction^37^. Each trial began with the appearance of a fixation point (FP) on the screen (Fig. 1a). Shortly after the monkey fixated the FP, two red dots appeared on the two sides of the monitor to indicate the two possible directions of motion in the trial (direction targets). After a short delay, the random dots stimulus appeared for 100–900 ms. Motion direction and strength (fraction of coherently moving dots) varied randomly from trial to trial. The random dots stimulus was followed by another delay. Then, on a random half of trials, a third target (called the sure target) appeared on the screen in the middle of this delay period. At the end of the delay, the FP disappeared, signaling the monkey to make its choice by making a saccadic eye movement to one of the targets. Choosing the correct direction target (right target for rightward motion and left target for leftward motion) resulted in a large reward (a drop of juice), whereas choosing the incorrect direction target resulted in a timeout. On the trials where the sure target was presented, the monkey could opt out of making a direction discrimination decision by choosing the sure target. The sure target was guaranteed to yield reward but the reward magnitude was smaller than that for the correct direction target (reward ratio, ~0.8).

We analyzed the data from the two monkeys separately. Monkeys M1 and M2 contributed 86,622 and 60,733 trials respectively to the dataset.

### Model fits

We used 10 ms time steps in our model fits and simulations because it offered a fine enough temporal resolution to explain the experimental data while keeping the computations manageable. All fits were based on maximum likelihood estimation (MLE). A detailed description of our model fitting procedure can be found in Section “Comparison of model predictions with experimental data” in the main text. We also tested the POMDP model with non-zero observation cost. This model, with two parameters (*w*_*z*_ and observation cost), was fit to the monkey’s choices on trials without the sure-bet target. Similar to the fitting procedure above for the zero-cost case, *σ*_0_ and *σ*_*z*_ were obtained by an iterative process that fit the average belief to average accuracy for each time step and *σ*_0_ was estimated based on the overall observation distribution. Because there is no closed-form equation for probability of choices in this model, we used grid search for the free parameters and estimated choice probability using particle filtering with 20,000 samples^70^. The grid resolution for cost was 10^−5^ while for *w*_*z*_, it was 0.01.

### One-step look-ahead search as the optimal strategy

For our results, we used one-step look-ahead search. Here we show that for an unbiased 2-alternative decision-making task such as ours, one-step look-ahead search results in the optimal POMDP policy for a non-decreasing observation cost over time. First, note that due to the symmetry of the task for direction choices, the optimal decision maker picks the choice with the highest belief. This means that when considering whether to terminate or continue acquiring observations, an optimal decision maker compares the observation cost and the resultant expected confidence (belief).

Second, the entropy, i.e. $-{b}_{right}{{{{{{\mathrm{log}}}}}}}\,({b}_{right})-{b}_{left}{{{{{{\mathrm{log}}}}}}}\,({b}_{left})$, has an inverse relationship with confidence. The expected information gain (i.e., decrease in entropy) decreases with more samples (here observations)^71^. As a result, the expected increase in confidence decreases with the number of observations as well. This means that if the expected increase in confidence with one more observation is less than the cost of the observation, the expected increase in confidence with *k* more observations is less than *k* times the cost of one observation. Thus, if the cost of observations is non-decreasing over time, comparing the expected confidence with the cost of an observation at the current time is enough to maximize the expected total reward. In other words, if the next observation is not worth its cost, making more observations would not be worth the cost either. Importantly, this holds for any observation function and state space as long as the probability distribution for observations does not change with time, which is true in our task (coherence does not change within a trial).

### Vuong’s statistical test

To test whether the monkey’s observation cost was negligible in our task, we used Vuong’s closeness test which compares the goodness of fit of two models, *u*_1_ and *u*_2_, based on their likelihood ratio and number of parameters^49^. With *N* data points in a data set, the *Z*-statistic of this test is:

11 $$Z=\frac{LR({u}_{1},{u}_{2})}{\sqrt{N}w}$$

where $LR({u}_{1},{u}_{2})={L}_{1}-{L}_{2}-0.5({K}_{1}-{K}_{2}){{{{{{\mathrm{log}}}}}}}\,N$. *L*_1_ and *L*_2_ are the log likelihoods, *K*_1_ and *K*_2_ are the number of parameters of *u*_1_ and *u*_2_, respectively, and *w* is the mean of the squares of the pointwise log-likelihood-ratios between the two models. We used Vuong’s test to compare the fits of the zero-cost and non-zero-cost POMDP models to our experimental data. There was no significant difference between the two models, even without penalizing the non-zero-cost POMDP model for having one more parameter (i.e., with *LR*(*u*_1_, *u*_2_) = *L*_1_ − *L*_2_).

### Simulations for increased stimulus variability

We used the POMDP model with parameters fit to Monkey M1’s data. For the low variability regime, the standard deviation of observations was *w*_*z*_ = 0.9 and the learned standard deviation was *σ*_*z*_ = 1.6. For the high variability regime, the standard deviation of observations was *w*_*z*_ = 1.5 without changing *σ*_*z*_ or any other parameter in the POMDP model.

### Simulations for simultaneous and sequential reports of choice and confidence

We used the following POMDP model parameters: *w*_*z*_ = 0.4, *σ*_*z*_ = 0.75, and *σ*_0_ = 5 with the 7 discrete coherence levels used in our monkey experiment and a constant observation cost of 2 × 10^−3^ per 10 ms to simulate the reaction-time task with 20,000 trials for each coherence. In the simultaneous report version of the model, both confidence and choice were calculated from the observations received prior to the model reaching its decision termination bounds. In the sequential report version, calculation of confidence continued to be influenced by observations during a 250 ms non-decision time after the choice.

### Exploring the effect of cost on sensitivity measurements and confidence report

We compared the POMDP model obtained from Monkey M1 with a model with similar parameters (*w*_*z*_, *σ*_*z*_, and *σ*_0_) but with an observation cost of 10^−4^ added to establish decision termination bounds in trials with coherence of 12.8% and duration of 400 ms. The confidence report was in the form of a binary rating (low or high) with a threshold of 0.63 applied to the belief about the choice. The sensitivity (${d}^{\prime}$) and meta-${d}^{\prime}$ were both 1.79 for zero observation cost. Increasing the cost to 10^−4^ decreased ${d}^{\prime}$ to 1.66 and meta-${d}^{\prime}$ to 1.23. We used 1 million samples to ensure the results were robust. The code from^30^ was used to calculate meta-${d}^{\prime}$. Slightly higher prior standard deviation (*σ*_0_ = 0.75) was used for better visualization of the effect in Fig. 6b. Qualitatively similar results are obtained for other motion coherence levels and durations.

### Prediction power of choice-congruent and choice-incongruent observations

First, we simulated the random dots motion discrimination task with one coherence (*w*_*z*_ = 1, *c* = 10.0%), one duration (800 ms) and a binary confidence rating of low or high with a POMDP model that had an exact model of the world (i.e., with the true *w*_*z*_ and *c*) but used the first *n* observations out of *t* = 80 (step size = 10 ms) observations. For each *n*, the confidence threshold was set to a value that made the probability of high confidence around 0.5.

To generate data points for Figures 7a, b, we trained logistic regression classifiers to predict the simulated choices and confidence ratings. Ten million trials were simulated for these analyses to ensure robust and accurate results. Our classifiers were implemented using the scikit-learn Python library^72^. For choice, the features of our classifier were the sum of positive observations and the sum of negative observations throughout each trial, including those beyond the first *n* samples used for simulating choice and confidence. For confidence, the features were the sum of choice-congruent observations and the sum of choice-incongruent observations throughout each trial. For the balanced classifier, to ensure balance of weights, we used a classifier with a feature consisting of the sum of all observations signed according to the choice (positive for choice-congruent and negative for choice-incongruent) as one feature.

For generating Figs. S2a and S2b, we repeated the above analysis with the same parameters but with the simulations using all *t* = 80 observations and the classifiers (representing the experimenter) using only *n* of those observations.

For Figs. 7c, d, S2c, and S2d we added zero-mean Gaussian noise with a standard deviation of 1.12 to the observations used in our classifiers to mimic the noisy estimate of a subject’s observations used by an experimenter.

### Reporting summary

Further information on research design is available in the Nature Research Reporting Summary linked to this article.

## Supplementary information

Supplementary Information

Reporting Summary

## Data Availability

The data analyzed in this study are available from R.K. (roozbeh@nyu.edu) upon reasonable request. Source data are provided with this paper.

## Source data

Source Data

## Code availability

All code supporting the findings of this study are available at: https://github.com/koosha66/POMDP-Confidence^73^.
