# Supplementary material for: Bayesian inference with incomplete knowledge explains perceptual confidence and its deviations from accuracy
Source: Nat Commun. 2021 Sep 29;12:5704. doi: 10.1038/s41467-021-25419-4 (PMC8481237; doi:10.1038/s41467-021-25419-4)
Supplement: Supplementary file 1 — Supplementary Information [file 41467_2021_25419_MOESM1_ESM.pdf]

Supplementary Information for: Bayesian Inference with Incomplete Knowledge Explains Perceptual Confidence and its Deviations from Accuracy

Koosha Khalvati, Roozbeh Kiani, Rajesh P. N. Rao

Trail-by-Trial Model Fitting compared to Batch Fitting

Our results are based on fitting our POMDP model parameters to data from all the trials taken together (batch estimation) as opposed to estimating the parameters iteratively in a trial-by-trial manner. This raises the following concern: does a trial-by-trial fitting process lead to a different estimate for σ_z and change the results?

To address this concern, we estimated model parameters iteratively for both monkeys M1 and M2 (see Figure 1a). Parameters were obtained based on maximum likelihood estimation with gradient descent. In the batch approach described in the main text, the gradient was calculated for all trials, and the optimization was performed in a few iterations. In the trial-by-trial approach, the gradient was calculated only for the current trial, which made the optimization process akin to stochastic gradient descent (SGD). To be more consistent with the subject's experience, each trial was used only for one iteration. We found that the results from SGD (or indeed gradient descent based on other unbiased sampling of the data set, e.g., mini batches) converged to the results from applying gradient descent on the entire dataset (Figure 1a). This is expected because of the large number of trials. Similar to the batch approach, SGD also produced $\sigma_z > w_z$, even when the initial values of σ_z and w_z were equal. Thus, an iterative approach leads to essentially the same conclusions as the batch approach.

Another possible concern is that we know neither the subject's true update strategy nor the true observations in each trial used for updating σ_z. However, it is reasonable to assume that similar to SGD, the subject increases σ_z (decreases overall confidence) when it receives negative feedback and decreases σ_z (increases overall confidence) when it receives positive feedback. The amount of update would depend on the actual observations in that trial (e.g., greater increase in σ_z when observations strongly indicating a particular choice result in negative feedback). Thus, although any trial-by-trial analysis is inherently limited by our lack of knowledge about the animal's true observations and update rule, we believe our SGD-based procedure is a reasonable approximation.

Finally, the data we analyzed were from the stable phase of data collection following extensive training with tens of thousands of trials with the same task structure. We therefore tested the effect of trial-by-trial estimation of model parameters after setting the initial value of parameters equal to the values from the batch approach, assuming that the subjects learned them during training. Figure 1b shows the evolution of σ_z and w_z with these more reasonable initial values, demonstrating the stability of these parameters across all trials in the experiment. Moreover, the evolution of overall accuracy in Figure 1c shows that this was not an artifact of the fitting process. As shown in the figure, σ_z and w_z fluctuated around the initial/batch values and were always within a 0.05 difference range. Accuracy was also quite stable (within a 1% difference range for monkey 1 and 3% for monkey 2).

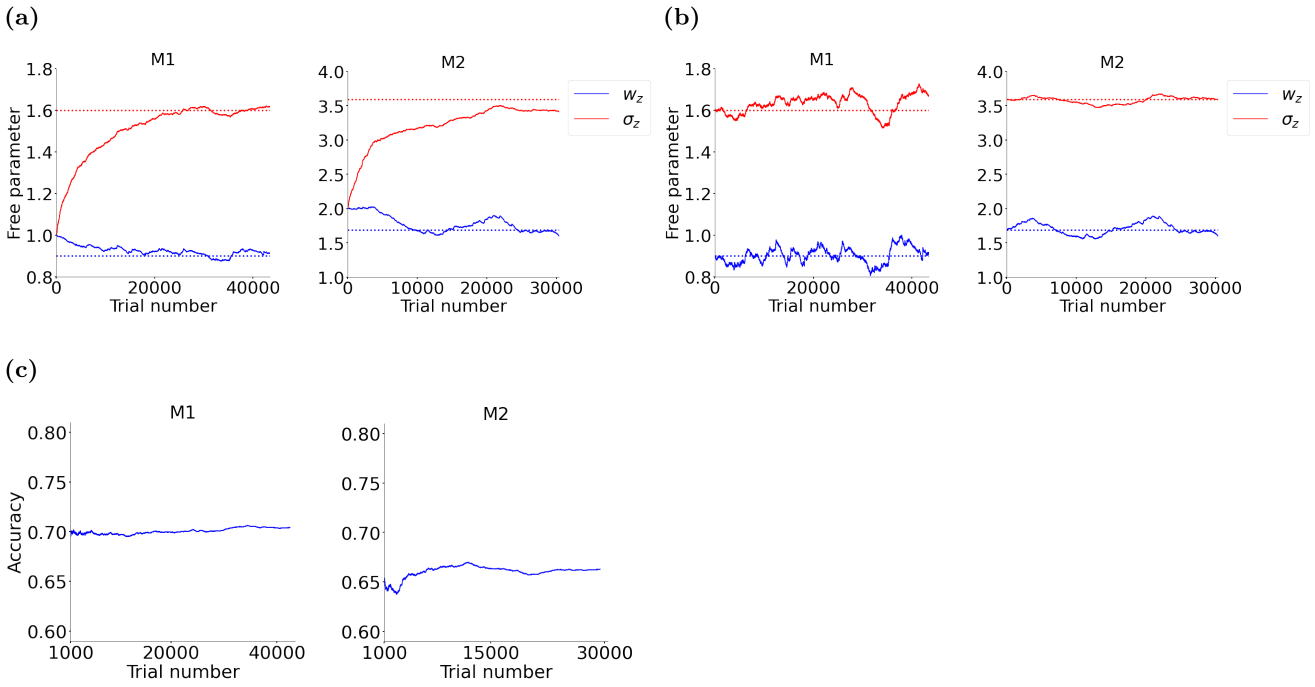

Supplementary Figure 1: Trial-by-trial model fitting results in similar parameter values as the batch approach. a) Because there are thousands of data points, parameters estimated from trial-by-trial (online) model fitting (red and blue lines) converge to similar parameters as the batch approach (dotted lines) at the end of the trials. b) Since our analyzed data is from the stable phase of data collection where the monkeys have already learned the task, it is more realistic to assume that the initial parameters are close to the parameters from the batch approach. For this more realistic initialization, the parameters remain within close range of the initial values throughout the entire experiment. c) Stability of overall accuracy indicates that this is not an artifact of our fitting process.

Sub-sampling the Decision Maker's Observations leads to Seemingly Higher Influence of Choice-Congruent Evidence on Confidence Ratings

In section 2.8, we demonstrated that when a decision maker uses a subset of available observations unbeknownst to the experimenter, the experimenter is prone to conclude a larger influence of choice-congruent observations on confidence. This artifact arises from misestimation of the congruent and incongruent evidence available to the decision maker at the time of decision. For similar reasons, if the experimenter analyzes only a subset of the observations used by the decision maker, they would artifactually find a larger effect of choice-congruent observations on confidence ratings. Figure 2 demonstrates this effect.

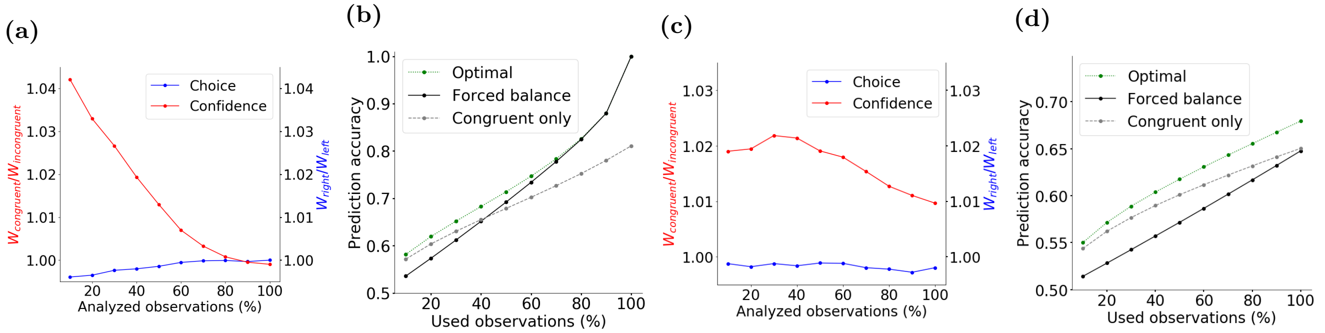

Supplementary Figure 2: Sub-sampling the decision maker's observations leads to seemingly higher influence of choice-congruent evidence on confidence ratings. Simulation parameters in this figure are similar to those in figure 7, except that the analyzed observations are a fraction of those used by the POMDP model. Note that the POMDP model that simulates the decision maker treats choice-congruent and incongruent observations identically. Therefore, the ground truth is equal weights for those observations, both for choice and confidence ratings. a) Choice-congruent observations gain larger weight in a classifier that predicts the decision maker's confidence (low or high) based on the observations analyzed by the experimenter. Note the change of y-axis scale compared to figure 7a, indicating that the weight imbalance here is much smaller than when the decision maker uses less evidence than the experimenter assumes. b) Similar to figure 7b. Forcing the classifier to assign identical weights to congruent and incongruent observations reduces classification accuracy. c-d) Same as a-b, but with noisy estimates of the decision maker's observations accessible to the experimenter. Such noise reduces the prediction accuracy of the confidence classifier, but more importantly, it also causes imbalanced weights in the optimal classifier that persist even when the classifier is based on all the observations used by the decision maker. Further, the contrast between the optimal classifier and the classifier with balanced weights increases compared to panel a. The noise comes from a zero-mean Gaussian distribution with a variance 25% larger than w_z^2, similar to figures 7c and 7d.
