# Supplementary material for: Bayesian inference with incomplete knowledge explains perceptual confidence and its deviations from accuracy
Source: Nat Commun. 2021 Sep 29;12:5704. doi: 10.1038/s41467-021-25419-4 (PMC8481237; doi:10.1038/s41467-021-25419-4)
Supplement: Supplementary file 2 — Reporting Summary [file 41467_2021_25419_MOESM2_ESM.pdf]

nature research

Corresponding author(s): Rajesh P.N. Rao

Last updated by author(s): Jul 13, 2021

Reporting Summary

Nature Research wishes to improve the reproducibility of the work that we publish. This form provides structure for consistency and transparency in reporting. For further information on Nature Research policies, see our Editorial Policies and the Editorial Policy Checklist.

Statistics

For all statistical analyses, confirm that the following items are present in the figure legend, table legend, main text, or Methods section.

n/a	Confirmed	
☐	☒	The exact sample size (*n*) for each experimental group/condition, given as a discrete number and unit of measurement
☐	☒	A statement on whether measurements were taken from distinct samples or whether the same sample was measured repeatedly
☐	☒	The statistical test(s) used AND whether they are one- or two-sided<br>*Only common tests should be described solely by name; describe more complex techniques in the Methods section.*
☐	☒	A description of all covariates tested
☐	☒	A description of any assumptions or corrections, such as tests of normality and adjustment for multiple comparisons
☐	☒	A full description of the statistical parameters including central tendency (e.g. means) or other basic estimates (e.g. regression coefficient) AND variation (e.g. standard deviation) or associated estimates of uncertainty (e.g. confidence intervals)
☒	☐	For null hypothesis testing, the test statistic (e.g. F, t, r) with confidence intervals, effect sizes, degrees of freedom and P value noted<br>*Give P values as exact values whenever suitable.*
☒	☐	For Bayesian analysis, information on the choice of priors and Markov chain Monte Carlo settings
☒	☐	For hierarchical and complex designs, identification of the appropriate level for tests and full reporting of outcomes
☒	☐	Estimates of effect sizes (e.g. Cohen's d, Pearson's r), indicating how they were calculated

Our web collection on statistics for biologists contains articles on many of the points above.

Software and code

Policy information about availability of computer code

Data collection	The data were collected using custom Matlab programs using Psychtoolbox and C programs using REX libraries.
Data analysis	Almost all of the code was written by the first author of the paper, Koosha Khalvati, in Python 3, Scikit-learn Python library (version 0.24). Publicly available code by Brain Maniscalco (http://www.columbia.edu/~bsm2105/type2sdt/) was also used.

For manuscripts utilizing custom algorithms or software that are central to the research but not yet described in published literature, software must be made available to editors and reviewers. We strongly encourage code deposition in a community repository (e.g. GitHub). See the Nature Research guidelines for submitting code & software for further information.

Data

Policy information about availability of data

All manuscripts must include a data availability statement. This statement should provide the following information, where applicable:

- Accession codes, unique identifiers, or web links for publicly available datasets
- A list of figures that have associated raw data
- A description of any restrictions on data availability

Data availability section has been included in the manuscript. The data analyzed in this study are available from R.K. (roozbeh@nyu.edu) upon reasonable request.

Field-specific reporting

Life sciences study design

All studies must disclose on these points even when the disclosure is negative.

Sample size	The data is from a published work, including two monkeys. One of the monkeys contributed 86,622 trials and the other contributed 60,733 trials. Using two monkeys with these numbers of trails are common in papers on perceptual decision making e.g. Roitman & Shadlen JNeuro 2002, and Churchland et al. Nature Neuroscience 2008.
Data exclusions	No data were excluded.
Replication	All of our results hold for both monkeys.
Randomization	There were no experimental groups in our study.
Blinding	There were no experimental groups in our study.

Reporting for specific materials, systems and methods

We require information from authors about some types of materials, experimental systems and methods used in many studies. Here, indicate whether each material, system or method listed is relevant to your study. If you are not sure if a list item applies to your research, read the appropriate section before selecting a response.

Materials & experimental systems

n/a	Involved in the study
☒	☐ Antibodies
☒	☐ Eukaryotic cell lines
☒	☐ Palaeontology and archaeology
☒	☐ Animals and other organisms
☒	☐ Human research participants
☒	☐ Clinical data
☒	☐ Dual use research of concern

Methods

n/a	Involved in the study
☒	☐ ChIP-seq
☒	☐ Flow cytometry
☒	☐ MRI-based neuroimaging
